# Changing environmental gradients over forty years alter ecomorphological variation in Guadalupe Bass *Micropterus treculii* throughout a river basin

**DOI:** 10.1002/ece3.4349

**Published:** 2018-07-30

**Authors:** Jessica E. Pease, Timothy B. Grabowski, Allison A. Pease, Preston T. Bean

**Affiliations:** ^1^ Texas Cooperative Fish & Wildlife Research Unit Texas Tech University Lubbock Texas; ^2^ U.S. Geological Survey Texas Cooperative Fish & Wildlife Research Unit Texas Tech University Lubbock Texas; ^3^ Department of Natural Resources Management Texas Tech University Lubbock Texas; ^4^ Heart of the Hills Fisheries Science Center Texas Parks and Wildlife Mountain Home Texas; ^5^ Present address: U.S. Geological Survey Hawaii Cooperative Fishery Research Unit University of Hawaii at Hilo Hilo Hawaii

**Keywords:** Centrarchidae, ecomorphology, flow alteration, intraspecific trait variation, urbanization

## Abstract

Understanding the degree of intraspecific variation within and among populations is a key aspect of predicting the capacity of a species to respond to anthropogenic disturbances. However, intraspecific variation is usually assessed at either limited temporal, but broad spatial scales or vice versa, which can make assessing changes in response to long‐term disturbances challenging. We evaluated the relationship between the longitudinal gradient of changing flow regimes and land use/land cover patterns since 1980 and morphological variation of Guadalupe Bass *Micropterus treculii* throughout the Colorado River Basin of central Texas. The Colorado River Basin in Texas has experienced major alterations to the hydrologic regime due to changing land‐ and water‐use patterns. Historical collections of Guadalupe Bass prior to rapid human‐induced change present the unique opportunity to study the response of populations to varying environmental conditions through space and time. Morphological differentiation of Guadalupe Bass associated with temporal changes in flow regimes and land use/land cover patterns suggests that they are exhibiting intraspecific trait variability, with contemporary individuals showing increased body depth, in response to environmental alteration through time (specifically related to an increase in herbaceous land cover, maximum flows, and the number of low pulses and high pulses). Additionally, individuals from tributaries with increased hydrologic alteration associated with urbanization or agricultural withdrawals tended to have a greater distance between the anal and caudal fin. These results reveal trait variation that may help to buffer populations under conditions of increased urbanization and sprawl, human population growth, and climate risk, all of which impose novel selective pressures, especially on endemic species like Guadalupe Bass. Our results contribute an understanding of the adaptability and capacity of an endemic population to respond to expected future changes based on demographic or climatic projection.

## 1 INTRODUCTION

Intraspecific variation among populations allows a species to adapt to a range of environments along natural gradients in temperature, elevation, altitude, or precipitation, and such variation has been documented in terrestrial and aquatic species. For example, latitudinal gradients in temperature have influenced natural variability in the body size of lizards, with larger individuals being found in lower latitude environments with warmer temperatures (Pincheira‐Donoso, Hodgson, & Tregenza, 2008; Zamora‐Camacho, Reguera, & Moreno‐Rueda, 2016). Plants have shown similar patterns across temperature gradients with increased metabolic rates, cell growth, and photosynthesis in warmer temperatures resulting in increased growth in species such as the flowering plant, *Arabidopsis thaliana* (Li, Suzuki, & Hara, 1998). However, anthropogenic disturbance has disrupted these natural gradients and established novel gradients along which populations must respond. Mechanisms of response vary from shifting ranges (Case & Taper, 2000), dispersal across ranges (Horváth, Vad, & Ptacnik, 2016; Kendall, Bjørnstad, Bascompte, Keitt, & Fagan, 2000), adaptation (Jackson & Colmer, 2005), or plasticity in trait response (Bell & Sultan, 1999; Morris, 2014). However, anthropogenic disturbances often occur more rapidly than the pace of these mechanisms.

Plasticity in a given trait may permit rapid population response to environmental stochasticity through matching the phenotype with the fluctuation in optimum fitness imposed by a disturbance (Charmantier et al., 2009; Chevin & Lande, 2010; S. Richter et al., 2012). Therefore, gaining information on intraspecific variation provides an understanding of the capacity of a species to respond to environmental fluctuation. This is especially important when vulnerability is heightened in restricted or fragmented habitats where dispersal or range shifts are not feasible mechanisms of response (Hodgson, Thomas, Dytham, Travis, & Cornell, 2012; McInerny, Travis, & Dytham, 2007). The prevalence of fragmentation and natural restrictions to the stream channel make aquatic species in rivers especially vulnerable (Braulik, Arshad, Noureen, & Northridge, 2014; Hugueny, Movellan, & Belliard, 2011). Running‐water habitats are restricted to mostly unidirectional natural gradients in abiotic and biotic influences within a dendritic network from headwaters to confluences with streams of increasing size (Fuller, Doyle, & Strayer, 2015; Junk, Bayley, & Sparks, 1989; Vannote, Minshall, Cummins, Sedell, & Cushing, 1980). Variability within a species is expected to optimize fitness along these environmental gradients, and the extent of variation dictates the range of local adaptations for populations within these freshwater systems (Hietpas, Bank, Jensen, & Bolon, 2013; Langerhans, 2009a).

Rapid, human‐induced modifications to river ecosystems, through changes in flow regime and land use, can influence the fitness of individuals leading to population‐level responses. These effects are usually assessed at relatively limited temporal and spatial scales; thus, it is not clear how basin‐wide alterations occurring across decades affect species with populations distributed across large basins. Spatially, intraspecific trait divergence has been identified for multiple fish species in comparisons between reservoir‐ and stream‐residing populations. For example, Black Shiner *Cyprinella venusta* individuals in reservoirs tended to have smaller heads, and deeper bodies in comparison with broader heads, and shallower bodies of stream individuals (Haas, Blum, & Heins, 2010). These traits are associated with occupying low current velocity habitats with high predator densities (Franssen, 2011; Haas et al., 2010). Increased predator evasion, swimming performance, and maneuverability for feeding are all associated with increasing body depth and caudal fin area suggesting such morphological shifts may be favored for individuals residing in reservoirs (Hambright, 1991; Holopainen, Aho, Vornanen, & Huuskonen, 1997; Langerhans, 2009b).

In addition to understanding of morphological divergence between reservoir and stream populations, there is a need to understand variation that may exist between populations separated by barriers to movement, such as in altered river networks. Population‐level information documenting the capacity of fish populations to adopt different morphologies in response to environmental change, as well as the consequences of these responses, assists in closing the gap in current understanding of population resiliency and identifies interpopulation differences critical to future management strategies. This is especially true for regions, such as central Texas, where human population growth has already strained on water supplies and climate projections are predicted to increase current extremes of temperature and precipitation resulting in prolonged drought and flooding conditions for which populations must respond (Jiang & Yang, 2012; Smith, David, Cardenas, & Yang, 2013). Understanding population responses to rapid environmental change induced by human perturbation in central Texas will provide insight on future population persistence under environmental variability.

One of the major waterways in central Texas is the Colorado River, which flows through the heavily urbanized area of Austin, Texas. Human populations are expected to increase drastically in the Colorado River Basin by 2050. For instance, the population in the Austin metropolitan statistical area (MSA) is projected to continue to grow at a rate greater than 30% and potentially reach 5 million people by 2050, in comparison the Austin MSA population was less than 600,000 in 1980 (Colby & Ortman, 2015; Hoque, McNeill, & Granato, 2014; The Office of the State Demographer, 2014). The mainstem Colorado River and its tributaries experience increasing urbanization and regulation as they flow into Austin. While the extent of urbanization declines as the river progresses downstream, the lower Colorado River below Austin is one of the most highly regulated stretches of river within the basin. Flow regime alterations throughout the basin due to urbanization are accompanied by agricultural diversions, irrigation return flows, and low water dams all of which lead to fragmentation and homogenization of instream habitat in the mainstem and tributaries of the Colorado River Basin. Variation in urbanization impacts throughout the Colorado River Basin provides an opportunity for determining the degree to which stream fishes exhibit plasticity in their behavior and biology in response to anthropogenic disturbance. The inclusion of strategies for monitoring intraspecific variation is emerging as an important consideration for successful management of populations (Mimura et al., 2017). Understanding and monitoring intraspecific variation helps to ensure the persistence of species, as well as the community framework and ecosystem function of the systems where these representative species reside. For instance, population‐level trait variation in Trinidadian guppies *Poecilia reticulata* altered ecosystem structure through impacts on algal and invertebrate densities, and the function of the ecosystem by influencing the primary production (Bassar et al., 2010). Guadalupe Bass *Micropterus treculii* is a species endemic to Texas and can be found throughout the Colorado River Basin along the present gradient of urbanization (Curtis, Perkin, Bean, Sullivan, & Bonner, 2015; Hendrickson & Cohen, 2015). Guadalupe Bass are considered fluvial habitat specialists, exhibiting both ontogenetic and seasonal shifts in habitat utilization. The vulnerability of populations to habitat alterations is heightened by the dependence of this species on instream structure and variable habitats for different life history stages. Ontogenetic habitat shifts occur throughout early life stages with movement toward increased current and depth following the juvenile stage (Edwards, 1980). The ability of Guadalupe Bass to respond and tolerate a range of conditions is evident based on the capacity of these populations to persist across a variety of habitats, as well as in novel environments under altered conditions. Previous research has found that intra‐population niche variation across nine Guadalupe Bass populations was mostly influenced by morphological variation. Individual specialization in wild Guadalupe Bass populations can occur at low levels of genetic diversity due to plasticity (Bean, 2012). Trophic diversity in Guadalupe Bass wild populations has been shown to be largely driven by plasticity in morphological characters in response to the differences in flows and productivity across systems (Bean, 2012). Further study of trait variation within Guadalupe Bass in response to changing environmental conditions would facilitate improved management and conservation of intraspecific variation.

Here, we examine the morphological variation across an environmental gradient over a 40‐year period throughout a large river basin using an archived range‐wide collection of Guadalupe Bass captured prior to major flow and land‐use alterations to compare to individuals collected under present‐day conditions. The objective of this study was to evaluate the effects of changing environmental gradients on ecomorphological variation in Guadalupe Bass populations across both temporal and spatial scales. Relationships between hydrologic alteration and landscape changes were compared to shape variation determined using geometric morphometric methods for Guadalupe Bass in the late 1970s and in contemporary conditions. Intensified landscape transitions are associated with flashier flow regimes and increased draw downs (Allan, 2004; Paul & Meyer, 2001). In such conditions, increased body depth in fish allows for increased maneuverability and initial speed, as opposed to more streamlined (fusiform) body shape, which reduces drag and increases endurance in conditions where there is sustained flow (Blake, 2004; Collar & Wainwright, 2009; Langerhans & Reznick, 2010). We expected morphological differentiation over time in relation to altered landscapes and hydrologic regimes to result in less fusiform body shape in contemporary populations. Measuring population‐level morphological response to anthropogenic changes across broad spatial and temporal scales allows for identification of plasticity within the species that has likely occurred with other physiological adjustments or adaptations through time. Understanding population‐level morphological responses to environmental stressors provides a baseline for management in urbanizing watersheds with increasing water withdrawals and land alteration.

## 2 MATERIALS AND METHODS

### 2.1 Study area

**Figure 1:**
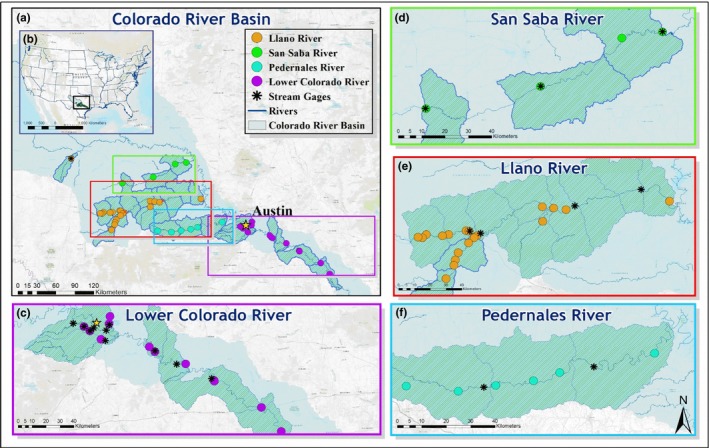
Map indicating the distribution of study sites and stream gages throughout the Colorado River Basin. Sites were chosen based on previous collections by Edwards (1980). Sites within each river system served as replicates representing the morphology of Guadalupe Bass within the mainstem and tributaries. Figures (a and b) indicate the location of the Colorado River in Texas and the distribution of the sites. Insets d–f indicate major tributary sites and the associated gages. Inset c shows sites and gages in the Austin, Texas area and located on the lower Colorado River below Longhorn Dam in Austin, Texas

The Colorado River Basin drains an area of 103,341 km^2^ and encompasses a large portion of Guadalupe Bass range. The majority (93,000 km^2^) of the basin lies within the karst ecoregion of the Edwards Plateau in central Texas where spring systems feed the Colorado's major tributaries: the Llano, Pedernales, San Saba, and Concho Rivers. We selected 30 sites corresponding to previous collections made by Edwards (1980) during 1975–1978. Sites spanned four different stream order categories: (a) three major tributaries (Llano, San Saba, and Pedernales), (b) smaller tributaries of the three major tributaries, (c) smaller tributary streams in the highly urbanized area of Austin, and (d) the mainstem lower Colorado River (Figure 1). Smaller tributaries in the upper watershed were classified as systems that were headwater streams originating with a stream order of 1 and not sharing a confluence with the mainstem Colorado River. The three major tributaries in the upper watershed were characterized as systems that shared a confluence with the mainstem of the Colorado River, and sites were located within a stream order greater than 4. Smaller tributaries that shared a confluence with the mainstem Colorado River within the boundaries of the Austin metropolitan area and originated with a stream order of 1 were considered smaller tributaries in the urbanized area of Austin. Morphometric collections for the mainstem Colorado only included sites on the lower Colorado River below the city of Austin, with a Strahler stream order of 7.

### 2.2 Environmental datasets

**Figure 2:**
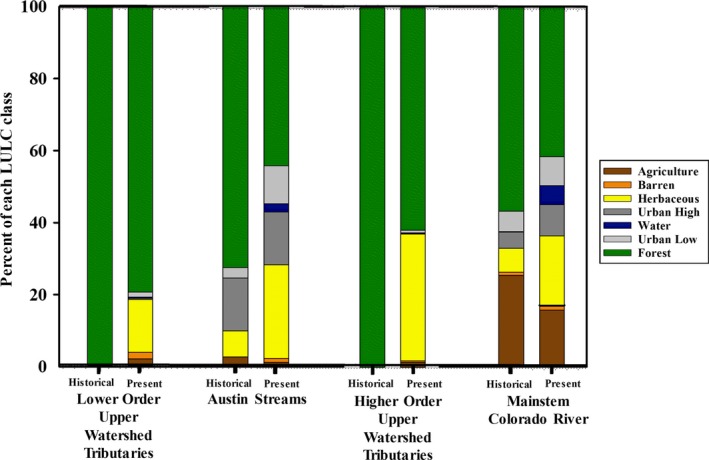
Bar graph indicating the changes in land use and land cover (LULC) within the sub‐watersheds in each of the four stream order classes between the historical period (1970–1980) and present time period (2012) The four separate stream order classes represent (a) headwater streams (Dove Creek, North Llano River, South Llano River, James River), (b) larger tributaries (San Saba River, Pedernales River, Llano River), (c) lower‐order Austin area streams (Barton Creek, Walnut Creek, Onion Creek), and (d) the mainstem Colorado River

Historical and present geospatial data were used to determine land‐use and land‐cover (LULC) changes for the 30 Hydrologic Unit Code Level‐10 (HUC‐10) watersheds (Supporting Information Table S1) which encompassed all study sites. Land‐use and land‐cover data from the 1970s and 1980s were obtained from the U.S. Geological Survey (USGS) National Water Quality Assessment (NAWQA) Program, which classified Landsat images (30‐m resolution) collected from 1972 to 1976 using the 45 class Anderson II classification system for LULC (McMahan, Frye, & Brown, 1984). Current LULC data at 10‐m resolution were obtained from the Texas Parks and Wildlife Department (TPWD) Ecological Systems of Texas (Diamond & Elliott, 2015), which used over 100 different LULC classes. Therefore, historical and present LULC data were reclassified into broad landscape classes of agriculture, barren, forested, herbaceous, water, urban high and urban low consistent between datasets in order to focus comparison on primary land conversion rather than vegetation types. Original LULC classes from both the historical and present datasets and the broader class within which each was reclassified into are defined in Supporting Information Table S2. The percentages of each LULC class within individual sub‐watersheds were quantified for both the historical and present datasets and used as the environmental variables for further analysis (Figure 2).

We evaluated changes in the variability of flow conditions across focal tributaries and the mainstem Colorado River between the 1970s and 1980s and present‐day collection periods. Hydrological alteration was determined from the historic and present discharge records using USGS stream gages closest to each sampling location (Supporting Information Table S3). Inter‐annual hydrologic variability within the periods was assessed using Indicators of Hydrologic Alteration (IHA) software (Richter, Baumgartner, Powell, & Braun, 1996) for four separate groups of hydrologic parameters: (a) monthly average, (b) annual extremes in minimum and maximum flow, and baseflow index (7‐day minimum flow/mean flow for year) (c) high and low pulse duration and frequency, and (d) overall change rate and frequency of water conditions. The coefficient of deviation (CD) for each of the hydrologic parameters was obtained for the given time period for further analysis. The CD measures the variability of individual hydrologic parameters as the (75th percentile–25th percentile)/50th percentile.

### 2.3 Morphometric measurements

**Figure 3:**
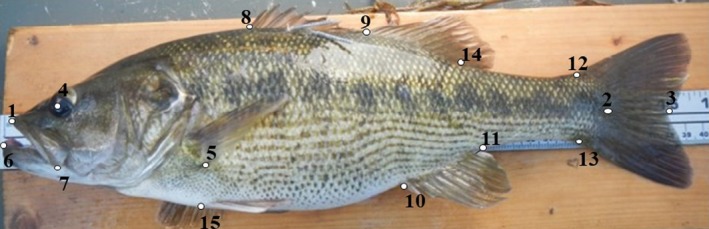
Location of the 15 landmarks used for morphological comparison of Guadalupe Bass *Micropterus treculii* throughout the Colorado River Basin, Texas. The 15 landmarks included in the analysis are as follows: (1) anterior edge of the premaxillary, (2) caudal peduncle, (3) fork of the caudal fin, (4) center of the eye, (5) the insertion of the last ventral ray on the pectoral fin, (6) anterior end of the dentary, (7) posterior‐most point of maxillary, (8) origin of first dorsal fin, (9) origin of the second dorsal fin, (10) origin of the anal fin, (11) insertion of last anal fin ray, (12) dorsal origin of the caudal fin, (13) ventral origin of the caudal fin, (14) insertion of the last ray of second dorsal fin, and (15) insertion of the pelvic fin

Guadalupe Bass (*n* = 348) were collected throughout Colorado River Basin from March 2014 to May 2016 using backpack electroshocking and seining. Boat electroshocking was used when applicable on the mainstem Colorado River. Each site was sampled twice each year. Guadalupe Bass were euthanized using a >400 mg/L aqueous solution of eugenol (Leary et al., 2013) and kept on ice until they were photographed. A digital camera (Nikon D3200, Melville, New York) was used to take a lateral left‐side photograph of each individual Guadalupe Bass collected during this study along with specimens (*n* = 457) collected during 1975–1978 and housed at the Texas Natural History Collection (see Supporting Information Table S4 for accession numbers). Due to concerns about potential preservation effects confounding comparisons of freshly‐caught and preserved fish (Berbel‐Filho, Jacobina, & Martinez, 2013; Gaston, Jacquemin, & Lauer, 2013; Sagnes, 1997), a subset of Guadalupe Bass was photographed then fixed and held in 10% formalin similar to the specimens collected by Edwards (1980). Photographs were taken with a reference scale. Landmarks, chosen based on previous fish morphological studies (Arbour, Hardie, & Hutchings, 2011; Franssen, Stewart, & Schaefer, 2013; Langerhans, 2008; Svanbäck & Eklöv, 2006), were digitized, and the scale was set using tpsDig v. 2 (Rohlf, 2004a). The 15 digitized landmarks were as follows: (1) anterior edge of the premaxillary, (2) caudal peduncle, (3) fork of the caudal fin, (4) center of the eye, (5) insertion of the last ventral ray on the pectoral fin, (6) anterior end of the dentary, (7) posterior‐most point of maxillary, (8) origin of first dorsal fin, (9) origin of the second dorsal fin, (10) origin of the anal fin, (11) insertion of last anal fin ray, (12) dorsal origin of the caudal fin, (13) ventral origin of the caudal fin, (14) insertion of the last ray of second dorsal fin, and (15) insertion of the pelvic fin (Figure 3). All photographs were marked by a single observer for consistency, and TPSUtil v. 1.46 (Rohlf, 2004d) was used to randomize images after a landmark had been marked on each photograph in order to prevent sequence effects.

Once morphometric images were landmarked, generalized Procrustes analysis (GPA) was used to account for the effects of translation, scale, and rotation on the spatial covariation of the landmarks using TPSRelw software (Rohlf, 2004c). TPSRelw was also used to calculate the square root of the sum of the squared distances from each landmark to the centroid for all 15 landmarks to determine centroid size, a metric for body size (Bookstein, 1984; Zelditch, Swiderski, Sheets, & Fink, 2004). While superimposition is useful for removing size differences in the shape variables, we also used centroid size as a covariate in further statistical analyses to account for allometric relationships between body size and shape differences (Elmer, Kusche, Lehtonen, & Meyer, 2010; Krabbenhoft, Collyer, & Quattro, 2009; Mitteroecker & Gunz, 2009; Webster & Sheets, 2010). Thin‐plate spline transformation grids were then used to visualize the individual variation in shape using TPSRegr software (Rohlf, 2004b).

### 2.4 Data analysis

Spatial and temporal morphological variation across all rivers was detected using mixed‐model multivariate analysis of covariance (MANCOVA) with 16 relative warps that explained 95.24% of the variance in the data as dependent shape variables (Hassell, Meyers, Billman, Rasmussen, & Belk, 2012; Kern & Langerhans, 2018). An *F*‐test based on Wilks's λ was used to determine statistical significance for all terms in the model with the exception of time period for the temporal model and river for the spatial model. Significance of these terms was determined from an *F‐*test that employed restricted maximum‐likelihood and the Kenward‐Rogers degrees of freedom adjustment in SAS using the MIXED procedure (Hassell et al., 2012; Sharpe, Langerhans, Low‐Décarie, & Chapman, 2015). The mixed procedure in SAS can effectively treat the population as a random effect, while also taking into consideration all relative warps at the same time. Following the methods of Hassell et al. (2012), we used an index variable to reflect the order of relative warps and treat the relative warps as repeated measures. Due to relative warps being treated as repeated measures on a single individual, individual and site within time period or river of origin were considered random variables in all models. Multivariate allometry was controlled for by including the centroid size, which is the square root of the sum of squared distance from each of the individual landmarks to the centroid, as a covariate. The partial variance explained by each factor in the model was estimated using an *F*‐test based on Wilks's *n* ^2^ (Langerhans & DeWitt, 2004).

To test for differences in body shape between historical and present‐day individuals, we modeled the main effects and interactions of time period and index variable with centroid size as a covariate. Interactions between the index variable and the main effect indicate the difference in shape for each of the relative warps independently and indicate morphological variation between the periods (following methods described in Wesner, Billman, Meier, & Belk, 2011; Hassell et al., 2012; Heinen‐Kay & Langerhans, 2013; Riesch, Martin, & Langerhans, 2013).

To test for spatial differences in body shape variation in contemporary individuals, we used river as a main effect, site nested within river as a random effect and log‐transformed centroid size as a covariate. We visualized morphological variation between time periods and spatially across rivers by deriving and eigenvector of divergence (*d*) for each of the terms. Eigenvectors of divergence were obtained from a principal component analysis (PCA) on the sum of squares and cross‐products matrix of the terms (Langerhans, 2009b). TPSRegr was used to generate thin‐plate spline deformation grids visualizing the shape variation along the divergence vector for the term of interest (Rohlf, 2004b).

Heterogeneity of slopes was significant in all models. Influence on the statistical models were checked following Kern and Langerhans (2018). The importance of the interaction between centroid size and main effect was less than that of the main effect in all models. The partial variance ($\eta_{p}^{2}$) ranged from 4.6% to 14.5%. The statistical significance of morphological variability between time periods and between rivers was not altered by the inclusion of the interaction. The interaction term was removed from all models due to the fact that inclusion did not alter the correlation of divergent vectors.

Discriminant function analysis (DFA) was used for cross‐validation and to determine assignment of individuals for river and time category based on size‐corrected shape variables (relative warps). To correct for size in shape variables, we used a MANCOVA with centroid size as the fixed factor and relative warps as the dependent variables and then retained the residuals as size corrected shape variables in the DFA. DFA was also carried out to determine classification into stream order classes, with the four classes as (a) headwater tributaries (Dove Creek, North Llano River, South Llano River, James River), (b) upper watershed tributaries (San Saba River, Pedernales River, Llano River), (c) urbanized Austin area streams (Barton Creek, Walnut Creek, Onion Creek), and (d) the mainstem Colorado River. Canonical correlation analysis (CCA) was used to determine variation in body shape distinguishing between historical and present individuals in relation to the most informative flow and LULC variables determined by stepwise discriminant function analysis. All analyses were performed using SAS 9.4 (SAS Institute, Inc., Cary, North Carolina). Data used in our analyses are available through the Dryad Digital Repository (https://doi.org/10.5061/dryad.0n52027).

## 3 RESULTS

### 3.1 Historical and present‐day land cover and land use in central Texas

During 1972–1976, the landscape within the HUC‐10 watersheds of the headwater tributaries (South Llano River, North Llano River, James River, and Dove Creek) and the upper watershed tributaries (San Saba River, Llano River, and the Pedernales River) of the Colorado River Basin was dominated by forested land cover of juniper, mesquite, oak savannahs, and scrub oak. Similarly, the lower‐order Austin urban watersheds (Barton Creek, Onion Creek, Walnut Creek) on the eastern edge of the Edwards Plateau ecoregion were dominated by forest land, in addition to 12%–20% urban area, and to a lesser degree herbaceous and barren land cover. The mainstem Colorado River downstream of the Edwards Plateau flows through the westernmost extent of longleaf pines, contributing to the high percentage of forested land cover historically within the watersheds within the East Central Texas Plains ecoregion. Watersheds in closest proximity to Austin, Texas encompassed 5% to 15% high‐intensity urban area, classified as greater than 70% impervious surface, and from 3% to 11% low‐intensity urban area, with less than 70% impervious surface (Diamond & Elliott, 2015; McMahan et al., 1984). Historically, the second‐most dominant land cover class in these watersheds was agriculture, with minimal amounts of barren or herbaceous land cover. In general, LULC changes in the Colorado River Basin since 1972–1976 were characterized by increased herbaceous land cover and decreased agriculture in increasingly urbanized watersheds. Similar transitions were documented between historical and present‐day LULC in the lower and higher‐order tributaries in the upper Colorado River Basin, as well as in lower‐order Austin streams and the lower mainstem Colorado River. All watersheds experienced a decrease in forested land cover and an increase in herbaceous land cover (Figure 2).

### 3.2 Hydrologic regime changes

Differences in hydrologic parameters between the two‐time periods were greatest in the lower‐order tributary systems in the upper Colorado River Basin and in the Austin urban streams (Supporting Information Figures S1 and S2). Variation between the two periods for lower‐order tributaries was related to decreases in the baseflow index and monthly summer flows and increases in the number of zero‐flow days and minimum flows. Differences in flow in Austin urban streams were related to increasing minimum flows as well as an increase in the number of zero‐flow days occurring per year. Additional differences between monthly flows were evident in Austin streams with present‐day flows in February, April, October, and December decreasing on average (−17%) compared to the historical time period, while present‐day flows were higher on average (21%) in June, July, September, and November. Lastly, baseflow index was greater than the mean in Austin streams during the present time period. Hydrologic changes in upper watershed rivers were related to increases in the baseflow index and monthly mean flows in August, and decreased minimum flows compared to historical regimes (Supporting Information Figures S1–S3). The mainstem Colorado River showed decreases in minimum flows and increases in maximum flows, as well as decreased flows in the late winter and early spring (Supporting Information Figure S4). Throughout the Colorado River Basin, differences in the hydrologic regimes between the two‐time periods indicated present‐day flows have increased flow variability, increased maximum flows, and diminished minimum flows. The small urban watersheds in Austin were an exception, with increasing average minimum flows, but the systems were flashier with more zero flow days (Supporting Information Figure S2).

### 3.3 Preservation effects

No effects of preservation in formalin on Guadalupe Bass morphology based on the landmarks used in the current study were found after 18 months (Supporting Information Figure S5). Although the study examining preservation effects was limited in duration relative to the length of time that fish collected by Edwards (1980) were held in formalin, previous studies have shown that preservation effects manifest relatively quickly after immersion in formalin (Jawad, 2003; Martinez, Berbel‐Filho, & Jacobina, 2013; Sagnes, 1997).

### 3.4 Spatial and temporal morphological variation

Centroid size had a significant effect on morphological scores ($\eta_{p}^{2}$ > 47%), indicating that RW scores and body size were correlated (Table 1). Time (i.e., whether the individual was collected between 1972 and 1980 as “historical” or between 2014 and 2016 as “present”) had the next strongest effect on morphological scores ($\eta_{p}^{2}$ = 25.23), indicating variability between the two‐time periods regardless of river of origin. There were consistent morphological differences associated with river between the two‐time periods ($\eta_{p}^{2}$ = 7.40). For example, relative warps for the mainstem Colorado River were significantly different between contemporary and historical individuals (*F*_4,61_ *=* 14.97, *p* < 0.01). Further, there were differences between rivers that were independent of time period ($\eta_{p}^{2}$ = 4.76; interaction between stream order and time period). Historical and present‐day Guadalupe Bass separated out along the first canonical axis (*F*_56,2799_ = 20.14, *p *<* *0.001), which was related to the placement of the pelvic and pectoral fin, and the distance between the premaxillary and maxillary. Contemporary individuals exhibited deeper bodies and a more anterior placement of the pectoral and pelvic fin origins relative to previously collected individuals. The morphological divergence between the contemporary and historical samples was sufficient to allow a DFA to correctly assign fish to their time periods 79% of the time using a single canonical dimension. The DFA based on river between the time periods was able to correctly assign historical individuals 48% of the time, while present individuals were correctly assigned to river of origin 26% of the time. The DFA based on stream order classes were able to correctly assign mainstem individuals 75%, major tributary individuals 74% of the time, urbanized Austin stream individuals 67% of the time, and the headwater tributary individuals 61% of the time.

**Table 1:** Results of MANCOVAs testing for body shape variation of contemporary Guadalupe Bass *Micropterus treculii* across all rivers and testing temporal shape variation. Time period indicates the difference between previously collected individuals (1975–1978) and individuals collected under current conditions (2014–2016)

Model	Model term	*F*	*df*	*p*	$\eta_{p}^{2}$ %
Spatial (only individuals collected from 2014–2016)	Log centroid size	21.01	16, 325	<0.0001	47.44
	Site(River)	1.97	576, 4,831	<0.0001	22.28
	River	3.04	160, 2,794	<0.0001	9.60
Temporal (individuals collected from 1975 to 1978 compared to individuals collected from 2014 to 2016)	Log centroid size	62.34	16, 705	<0.0001	58.59
	Time period	114.87	16, 705	<0.0001	25.23
	River	3.56	160, 6,039	<0.0001	7.40
	River × time period	2.21	64, 2,762	<0.0001	4.76

### 3.5 Environment and morphological divergence

**Figure 4:**
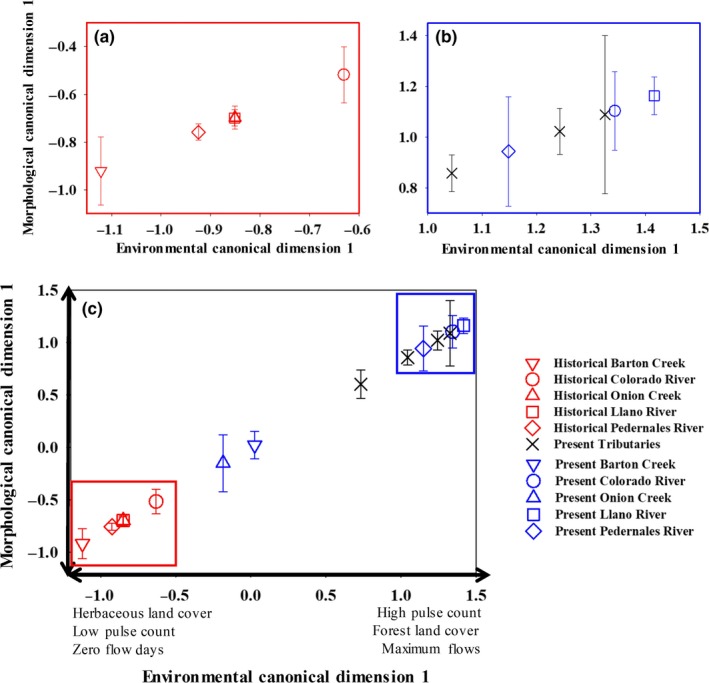
Mean morphological scores for historical and present‐day collected Guadalupe Bass *Micropterus treculii* where red symbols represent historical morphological canonical scores and blue symbols represent present morphological canonical scores for the mainstem Colorado River and tributaries of the Colorado River. Historical specimens were obtained from Texas Natural Historical Museum for morphological analysis. Tributaries where historical specimens were not archived or available are indicated by black X's. The mainstem Colorado River and the three tributaries for which there were museum specimens are indicated by similar symbols with historical means represented in red and present means represented in blue. The first environmental canonical dimension representing hydrological and percentage difference in landscape is shown on the *X* axis. Insets a and b show the most closely related rivers from c

Morphological and environmental variables were related along two canonical functions for Guadalupe Bass over the 40‐year time period. Morphological divergence between present and historical individuals was predicted by the first canonical variate (*R*_c_ = 0.81, *F*_88,2493.7_ = 13.31, *p *<* *0.001) with 67.0% of the variation in morphology being explained by the first environmental variate (Figure 4). The first morphological canonical variate primarily separated individuals based on the placement of the pelvic and pectoral fin, indicating body depth, and the distance between the premaxillary and maxillary, which is a measure of the head shape (Supporting Information Table S5). The first environmental variate of the canonical correlation analysis was associated with the increases in herbaceous land cover, decreases in forested land cover, and increasing maximum flows and the low pulse count (Supporting Information Table S6). Onion Creek and Barton Creek, two urban streams, were the only exceptions to these trends with individuals under present conditions in both creeks separating out along the first canonical function gradient of morphological variation with historical individuals (Figure 4). All other present‐day Guadalupe Bass tended to have increased body depth associated with a shift in the placement of the pelvic fin under increased maximum flow conditions. In comparison, historical individuals tended to have shallower body depths, with forested land cover and the number of low pulses contributing to the environmental canonical variate.

**Figure 5:**
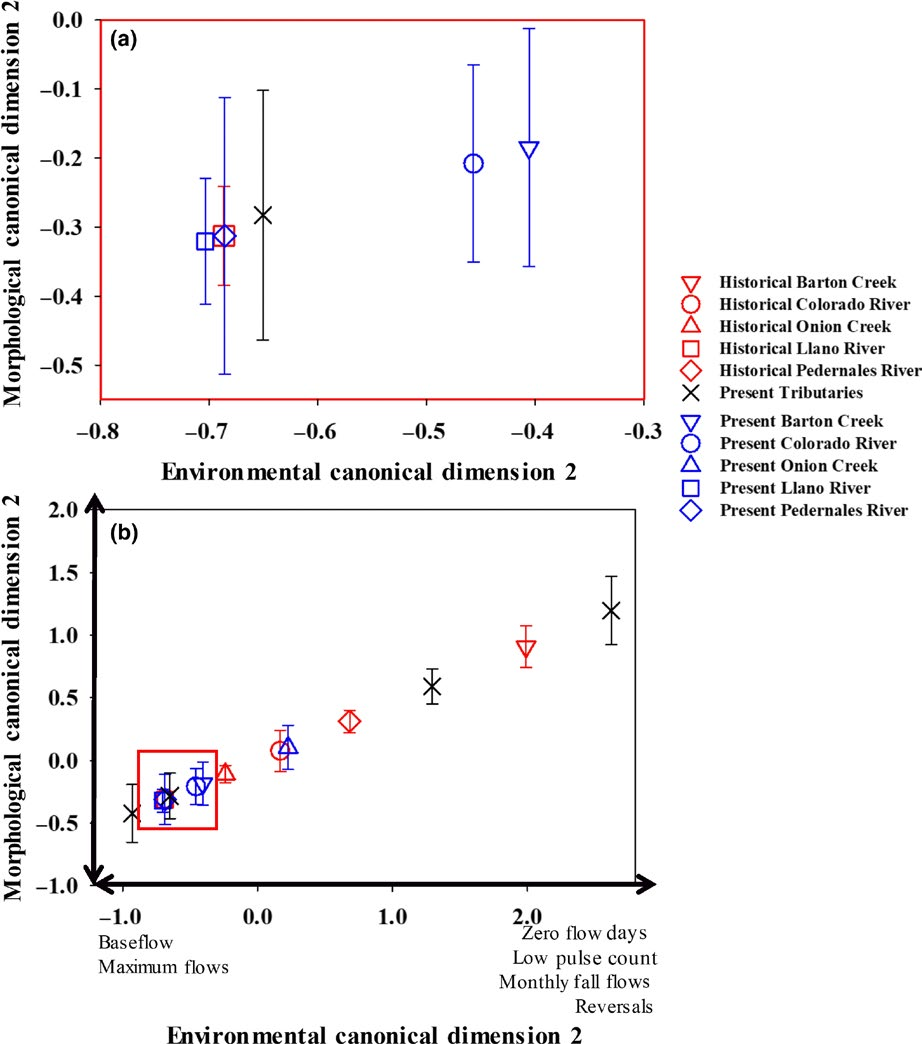
Mean morphological scores for historical and present‐day collected Guadalupe Bass *Micropterus treculii* where red symbols represent historical morphological canonical scores and blue symbols represent present morphological canonical scores for the mainstem Colorado River and tributaries of the Colorado River. Historical specimens were obtained from Texas Natural Historical Museum for morphological analysis. Tributaries where historical specimens were not archived or available are indicated by black X's. The mainstem Colorado River and the three tributaries for which there were museum specimens are indicated by similar symbols with historical means represented in red and present means represented in blue. Environmental canonical scores representing hydrological and percentage difference in landscape are shown on the *X* axis. Inset a shows the most closely related sites from b

**Figure 6:**
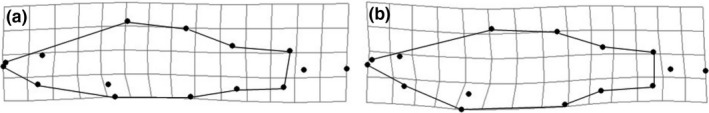
Thin‐plate spline transformation grids illustrating the morphological variation in body shape between historical (a; pre‐1980) and present‐day (b; post‐2012) Guadalupe Bass *Micropterus treculii*. Transformation grids are magnified 3× to better visualize the differences

The second morphological canonical variate represented morphological variation amongst and within sites throughout the Colorado River Basin (Figure 5). Differences were largely related to the distance between the caudal fin and anal fin, which represented a change in the length, as well as a shift dorsally of the caudal fin representing a change in depth (Figure 6). The main predictors loading the second environmental canonical variate were number of zero‐flow days, baseflow index, low pulse count, thirty‐day maximum flows, and monthly flows in the fall. Present‐day Guadalupe Bass showed an increased distance between the anal fin and the caudal fin. However, contemporary individuals collected in Onion Creek exhibited shorter distance between the anal fin and the caudal fin, more similar to individuals collected historically from Barton Creek.

## 4 DISCUSSION

The Colorado River Basin has experienced a range of changes in LULC and flow conditions over the past four decades, and these changes were associated with temporal morphological shifts in Guadalupe Bass populations. Morphological differentiation between the time periods showed a gradient in body depth with historical individuals being largely distinguished based on their shallower bodies compared to present‐day, deeper‐bodied Guadalupe Bass. In general, deeper bodied Guadalupe Bass in the contemporary, altered Colorado River Basin experienced more variable flows with higher maximum flows and lower minimum flows in comparison with historical flow conditions. These flashier flow regimes, characterized by short duration and high magnitude flow events, are commonly associated with urban areas (Konrad & Booth, 2005; Paul & Meyer, 2001; Walsh et al., 2005) due to increased impervious surface, preventing water infiltration and increasing surface runoff (Shuster, Bonta, Thurston, Warnemuende, & Smith, 2005; Yang, Bowling, Cherkauer, & Pijanowski, 2011).

While there is a well‐established link between fish morphology and water velocity, the variability in the impact that urbanization and anthropogenic alteration of the landscape has on flow patterns through time has been shown to result in varying morphological responses (Franssen et al., 2013; Istead, Yavno, & Fox, 2015; Leavy & Bonner, 2009). Blacknose dace *Rhinichthys obtusus* in urbanized streams of North Carolina had more streamlined morphology, whereas Creek Chub *Semotilus atromaculatus* under the same environmental conditions showed deeper body morphology (Kern & Langerhans, 2018). In the Colorado River in Texas, we found that Guadalupe Bass exhibited increased body depth in response to changes in hydrologic patterns associated with increased urbanization, as well as increased herbaceous cover in more rural watersheds. In the mainstem Colorado and Austin stream watersheds, there was an increase in urbanized area likely contributing to the increase in variability in these flow regimes. However, the increased body depth of contemporary individuals residing in more rural upper watershed tributaries is potentially due to the transition of forested land to other cover types resulting in similar patterns of hydrologic change. Changes in LULC outside of these highly urbanized areas primarily involved loss of forest land cover and increasing herbaceous land cover. Increases in herbaceous land cover is likely due to the transition of forested land cover to grazing area for livestock (Paukert, Pitts, Whittier, & Olden, 2011), one of the primary land uses within the Colorado River Basin. Although herbaceous land cover is often associated with benefits of infiltration and decreased surface runoff, the grazing of livestock can cause soil compaction, which acts similarly to an impervious surface (Chyba, Kroulík, Krištof, Misiewicz, & Chaney, 2014; Hamza & Anderson, 2005). Deeper bodied individuals may be favored under these patterns in hydrological alteration associated with these landscape changes throughout the basin, due to the morphological advantages that increased body depth has on the maneuverability and increased bursts in swimming speed (Webb, 2006). Under decreased current or stagnant environments deeper bodied individuals have shown improved foraging ability and predator avoidance performance (Franssen et al., 2013; Santos & Araújo, 2015). For example, when Bluegill *Lepomis macrochirus* and Green Sunfish *Lepomis cyanellus* were compared between lotic and lentic habitats, which naturally represent two extremes in flow conditions, the individuals in the reservoir habitats tended to have deeper bodies, while streamlined individuals were found in lotic environments (Gaston & Lauer, 2015). Morphological differentiation in Guadalupe Bass was not compared across the extremes of stream versus reservoir habitat; however, similar increases in body depth were observed between contrasting temporal flow conditions.

The observed trait changes were consistent with morphological variation observed in other fish species across spatial environmental gradients, but to our knowledge, such changes have rarely been identified over long temporal scales. Previous studies assessed spatial trait variation in fish populations between contrasting environmental conditions, such as the absence of a predator (Holopainen et al., 1997; Robinson, Januszkiewicz, & Koblitz, 2008) or between a lotic and lentic flow regime (Franssen, 2011; Franssen & Tobler, 2013). However, assessing temporal trait variability is challenging and often relies on extensive historical collections. Access to historical collections prior to rapid human‐induced change presented the unique opportunity to study the response of populations to varying environmental conditions through space and time. Our results along with those of Kern and Langerhans (2018) suggest that anthropogenic alteration has the ability to alter fish morphology, and this continued environmental change could impact ecosystem structure and function (Bassar et al., 2010; Crutsinger, 2016). These results contribute to an understanding of trait variation that may help to buffer populations under conditions of increased urbanization and sprawl, human population growth, and climate risk, all of which impose novel selective pressure on species (Nelson et al., 2009; Reed, Waples, Schindler, Hard, & Kinnison, 2010), especially endemic species like Guadalupe Bass (Kwon et al., 2012; McDonald et al., 2011).

The sensitivity of the Colorado River Basin to the changing climate combined with the narrow range and population declines of Guadalupe Bass associated with fragmentation and hybridization with Smallmouth Bass necessitates investigating the ability of trait variation to buffer the Guadalupe Bass population (Bean, Lutz‐Carrillo, & Bonner, 2013; Curtis et al., 2015; Koppelman & Garrett, 2002; Littrell, Lutz‐Carrillo, Bonner, & Fries, 2007). In central Texas, the persistent drawdown on the Ogallala Aquifer is currently occurring at an unsustainable rate (Vaughan et al., 2012). Increased population growth accompanied by climatic changes throughout the state of Texas have already reduced the amount of water available (Liu et al., 2012; Smith et al., 2013). Additional increases in temperature and decreased precipitation expected for the region will increase the demand for water abstraction and storage (Chin, Laurencio, & Martinez, 2008; Yin, Yang, & Petts, 2012). Continued anthropogenic alteration of flow regime will impact Guadalupe Bass and further drive variability in morphology. Understanding the limitations of intraspecific variability to buffer these populations will be crucial to future management under increased demographic and climatic changes (Garrett, Birdsong, Bean, & McGillicuddy, 2015; Mimura et al., 2017).

In addition to showing variable body depth, Guadalupe Bass also showed underlying spatial morphological variation in the caudal peduncle. There may be two possible mechanisms for these morphological differences across the Colorado River Basin. Firstly, a stouter and deeper caudal peduncle may permit an individual to remain in position (de Assumpção et al., 2012), especially under higher maximum flows and longer duration of high pulses. However, under contemporary conditions of decreased baseflow index, there may also be deeper and stouter caudal fins for maneuverability and increased foraging efficiency. The caudal peduncle plays a major role in fish movement and maneuverability, and both of these mechanisms have been shown to drive morphological variation (Imre, McLaughlin, & Noakes, 2002; McLaughlin & Grant, 1994). For example, in Brook Charr *Salvelinus fontinalis* high‐velocity flows have been shown to increase the caudal fin height and depth of the caudal peduncle (Hendry et al., 2010; Imre et al., 2002; Istead et al., 2015); however, high‐velocity flows have also been shown to favor slender narrower caudal peduncles with deeper caudal peduncles being favored inlow‐velocity flows (Blake, 2004; Langerhans & Reznick, 2010; Vogel, 1994).Understanding how Guadalupe Bass are changing in response to anthropogenic habitat disturbances throughout a basin provides an indication of the resiliency of this species and reveals issues that may impede future conservation and management efforts. When implementing restoration, consideration for population‐specific responses to unique environmental stressors may also be crucial when managers are choosing the proper broodstock to combat introgression or repatriate populations. For example, stocking lower Colorado River individuals in the Pedernales River may not be as successful or result in similar recruitment success as if San Saba River broodstock fingerlings were stocked in the Pedernales river. Incorporating intraspecific variation into management efforts will assist managers in the continued effort to combat introgression and inform restoration efforts that are paired with the pace of phenotypic response time (e.g., Ensslin, Tschöpe, Burkart, & Joshi, 2015). Discerning the morphological response of species will allow managers to modify timing and efforts for populations that have adapted under altered environments, thus increasing the success of these management efforts.

The patterns in morphological variability of Guadalupe Bass in response to changes in the flow regime, whether natural or anthropogenic, suggest that intraspecific trait variation may support resilience of populations under fluctuating conditions. Population adaptive capabilities have been shown to enhance fitness under differing environmental demands (Laughlin & Messier, 2015; Reed et al., 2010). However, there are potentially a multitude of other environmental or interspecific interactions driving trait change in Guadalupe Bass, such as predation (Eklöv, Svanbak, Eklo, & Svanba, 2006; Hendry, Kelly, Kinnison, & Reznick, 2006), habitat use (Brinsmead & Fox, 2002; Langerhans, Layman, Langerhans, & Dewitt, 2003), and diet (O'Neill & Gibb, 2014; Reimchen & Nosil, 2002; Ward‐Campbell, Beamish, & Kongchaiya, 2005). Our data do not permit us to determine the precise drivers, or whether observed intraspecific variation is due to genetic or phenotypic variation. However, we do show that population‐level variation has possibly contributed to the persistence of Guadalupe Bass throughout the Colorado River Basin. Further study is warranted to determine if the trait differences we observed between tributary and mainstem populations are contributors to variable fitness of individuals and affect further population tolerance and dynamics. Persistence and resiliency of populations under hydrologic alteration and transitioning landscapes have also been seen in other aquatic and terrestrial animal populations (Craven, Peterson, Freeman, Kwak, & Irwin, 2010; Goodman, Miles, & Schwarzkopf, 2008; Kolbe, Lockwood, & Hunt, 2011; Ribera, Doledec, Downie, & Foster, 2001). In contrasting flow conditions between intermittent and permanent streams, the alpine caddisfly *Allogamus uncatus* exhibits plasticity in life‐history traits related to growth and emergence that allowed *A. uncatus* to persist in streams where drying might occur multiple times a year (Shama & Robinson, 2006, 2009). Additionally, population resilience across landscapes has been observed in association with variation in morphological traits related to flying for female speckled wood butterfly *Pararge aegeria* under conditions of land cover transition from woodland to agriculture. *Pararge aegeria* females developed increased total dry mass and wing loading when offspring were transplanted to agricultural landscapes in comparison to woodland (Merckx & Dyck, 2006). Population resilience and persistence can become evident on a generational timescale in some organisms. Therefore, understanding the intraspecific variability enabling a population to maintain their distributional range is crucial as landscapes and flow regimes continue to undergo alteration disrupting the natural environmental gradients that originally established variation in population‐level traits. For that reason, understanding the capacity of a population to persist due to intraspecific trait variation has implications in modern conservation that need to be addressed.

Environments naturally fluctuate; consequently, populations are never really at equilibrium due to the stressors imposed by the environment. If populations are continually driven in one direction by environmental change from anthropogenic influence, they may no longer have the adaptability and capacity to respond to future changes expected based on demographic or climatic projections. For example, desert‐adapted spadefoot tadpole populations have developed accelerated metamorphosis to avoid desiccation, but continual pond drying has diminished the plasticity in metamorphosis timing of the desert population, in comparison with nondesert populations (Gomez‐Mestre & Buchholz, 2006; Kulkarni, Gomez‐Mestre, Moskalik, Storz, & Buchholz, 2011). The preservation of variation within a population fortifies the species’ ability to respond to environmental change and promotes persistence of populations in areas that are heavily disturbed or projected to have increased alteration. Continued research on species and population tolerance through the study of phenotypic plasticity will provide further information on the conservation of communities with impeding anthropogenic alteration, and natural environmental fluctuations (Geist, 2011; Hendry et al., 2011).

## CONFLICT OF INTEREST

None declared.

## AUTHOR CONTRIBUTIONS

Dr. Timothy Grabowski, Dr. Preston Bean, and Dr. Allison Pease contributed to the design and conception of the project. Data collection and acquisition was performed by Jessica Pease, Dr. Bean, and Dr. Grabowski. Jessica Pease, Dr. Grabowski, and Dr. Pease conducted the data analysis and interpretation of the results. The manuscript was drafted, revised, and approved by all authors. All authors ensure the accuracy and integrity of this research and accept accountability for these results. Data used in our analyses are available through the Dryad Digital Repository (https://doi.org/10.5061/dryad.0n52027).
