# Supplementary material for: Changing environmental gradients over forty years alter ecomorphological variation in Guadalupe Bass Micropterus treculii throughout a river basin
Source: Ecol Evol. 2018 Jul 30;8(16):8508–22. doi: 10.1002/ece3.4349 (PMC6145027; doi:10.1002/ece3.4349)
Supplement: Supplementary file 1 [file ECE3-8-8508-s001.docx]

Supporting Information

**Changing environmental gradients over forty years alter ecomorphological variation in Guadalupe Bass *Micropterus treculii* throughout a river basin**

Jessica E. Pease^1^, Timothy B. Grabowski^2^^[[1]](#footnote-1)^*, Allison A. Pease^3^ and Preston T. Bean^4^

*^1^Texas Cooperative Fish & Wildlife Research Unit, Texas Tech University, Lubbock, Texas 79409; (806) 742-2851; Email:* [*jessica.pease@ttu.edu*](mailto:jessica.pease@ttu.edu)

^2^*U.S. Geological Survey, Texas Cooperative Fish & Wildlife Research Unit, Texas Tech University, Lubbock, Texas 79409; (808) 932-7575; Email: tgrabowski@usgs.gov*

^3^*Department of Natural Resources Management, Texas Tech University, Lubbock, Texas 7940; (806) 742-2841; Email: allison.pease@ttu.edu*

*^4^Heart of the Hills Fisheries Science Center, Texas Parks and Wildlife, Mountain Home, Texas, 78058; (830) 866-3040; Email: preston.bean@tpwd.texas.gov*

Table S1: Summary table of site locations and the associated ten-digit hydrologic unit code (HUC) and United States Geological Survey stream gage number.

| **Site** | **River** | **River** | **Latitude** | **Longitude** | **HUC 10** | **USGS Gage** | **Watershed** |
| --- | --- | --- | --- | --- | --- | --- | --- |
| Barton Creek Greenbelt S Capital of Texas Hwy 360 | 1 | BART | 30.244703 | -97.8022 | 1209020503 | 8155240 | 22 |
| Lost Creek Access to Barton Creek Greenbelt off Lost Creek Blvd. | 1 | BART | 30.2743 | -97.8444 | 1209020503 | 8155240 | 22 |
| South of US 90 Alt Boat Ramp in Eagle Lake | 2 | COLO | 29.5532 | -96.4008 | 1209030201 | 8161000 | 30 |
| South of Fisherman's Park Boat Launch in Bastrop | 2 | COLO | 30.0994 | -97.3211 | 1209030102 | 8159200 | 27 |
| South of Webberville Park | 2 | COLO | 30.2019 | -97.4868 | 1209030102 | 8159200 | 27 |
| North of Fisherman's Park Boat Launch in Bastrop | 2 | COLO | 30.1333 | -97.3614 | 1209030102 | 8159200 | 27 |
| South of the Fannin St. TX 71 Business Boat Ramp | 2 | COLO | 29.7158 | -96.5416 | 1209030107 | 8160400 | 29 |
| State Highway 71 | 2 | COLO | 29.9012 | -96.8870 | 1209030107 | 8160400 | 29 |
| South of State Highway 71 Boat Ramp | 2 | COLO | 29.8957 | -96.8843 | 1209030107 | 8160400 | 29 |
| South of the State Hwy 71 crossing in Smithville | 2 | COLO | 30.0145 | -97.0942 | 1209030104 | 8159200 | 28 |
| FM 2335 outside Knickerbocker | 3 | DOVE | 31.2738 | -100.6308 | 1209010203 | 8130500 | 3 |
| James River Rd. crossing north of Eckert James River Bat Cave Preserve | 4 | JAME | 30.5725 | -99.3233 | 1209020404 | 8150700 | 17 |
| RR 1871 | 5 | LLAN | 30.6579 | -99.3246 | 1209020402 | 8150000 | 16 |
| RR 2389 James River and Llano River confluence. | 5 | LLAN | 30.6502 | -99.2507 | 1209020405 | 8150700 | 17 |
| South Llano and North Llano confluence off Camino Rio St. | 5 | LLAN | 30.4926 | -99.7567 | 1209020202 | 8148500 | 15 |
| FM 3404 in Kingland, Texas | 5 | LLAN | 30.6824 | -98.4835 | 1209020408 | 8151500 | 21 |
| Simonsville Rd. | 5 | LLAN | 30.64002 | -99.168097 | 1209020405 | 8150700 | 18 |
| Bear Creek at Interstate 10 | 6 | NLR | 30.5210 | -99.8293 | 1209020203 | 8148500 | 12 |
| CR260 | 6 | NLR | 30.4986 | -100.0927 | 1209020203 | 8148500 | 11 |
| CR271 | 6 | NLR | 30.5181 | -99.8102 | 1209020203 | 8148500 | 12 |
| CR274 | 6 | NLR | 30.4981 | -99.9448 | 1209020203 | 8148500 | 12 |
| CR275 | 6 | NLR | 30.4909 | -99.9864 | 1209020203 | 8148500 | 11 |
| CR260 at River Rd in Roosevelt | 6 | NLR | 30.4796 | -100.1195 | 1209020203 | 8148500 | 11 |
| CR310 to River Rd. in Sonora | 6 | NLR | 30.4826 | -100.1474 | 1209020203 | 8148500 | 11 |
| McKinney Falls State Park | 7 | ONION | 30.1885 | -97.7205 | 1209020504 | 8159000 | 23 |
| RR 1320 | 8 | PEDE | 30.2726 | -98.5455 | 1209020602 | 8153500 | 25 |
| Texas Hwy 16 | 8 | PEDE | 30.2070 | -98.9790 | 1209020601 | 8153500 | 24 |
| Hamilton Pool Rd. (FM 3238) Crossing | 8 | PEDE | 30.3399 | -98.1392 | 1209020504 | 8152900 | 26 |
| U.S. Route 290 | 8 | PEDE | 30.2276 | -98.8188 | 1209020602 | 8153500 | 25 |
| Fiedler Rd. | 8 | PEDE | 30.2277 | -99.2001 | 1209020601 | 8152900 | 24 |
| RR 1623 | 8 | PEDE | 30.2433 | -98.6572 | 1209020602 | 8153500 | 25 |
| Decker St. off TX Hwy 83 in Menard | 9 | SABA | 30.9190 | -99.7840 | 1209010905 | 8144500 | 8 |
| FM 340 / S. Cotton Belt Rd. | 9 | SABA | 31.1910 | -98.9026 | 1209010908 | 8146000 | 10 |
| Texas Hwy 16 at the intersection with FM 1480 | 9 | SABA | 31.2133 | -98.7199 | 1209010908 | 8146000 | 10 |
| US Hwy 377/87 outside Brady | 9 | SABA | 31.0040 | -99.2695 | 1209010907 | 8146000 | 9 |
| Flatrock Ln. off US 377 | 10 | SLR | 30.4790 | -99.7779 | 1209020302 | 1111111 | 14 |
| CR150 | 10 | SLR | 30.3936 | -99.8820 | 1209020302 | 1111111 | 14 |
| CR408 | 10 | SLR | 30.2419 | -99.9628 | 1209020302 | 1111111 | 13 |
| US Hwy 377 at the first crossing south of Junction | 10 | SLR | 30.3504 | -99.9017 | 1209020302 | 1111111 | 14 |
| South Llano State Park (State Park Rd. 73) | 10 | SLR | 30.4502 | -99.8128 | 1209020302 | 1111111 | 14 |
| Stevenson RR in Telegraph | 10 | SLR | 30.3199 | -99.9109 | 1209020302 | 1111111 | 14 |
| Springdale Rd. | 11 | WC | 30.3375 | -97.6502 | 1209020503 | 8158600 | 22 |
| Walnut Creek Bike Trail off FM 969 | 11 | WC | 30.2906 | -97.6567 | 1209020503 | 8158600 | 22 |

Table S2: Summary table of Hydrologic Unit Codes for Sub-watersheds (Figure 1) encompassing study sites of interest. HUC 10 watersheds were accessed through USGS.

| **SUBBASIN** | **HUC_10** | **HU_10_NAME** | **Area_km** |
| --- | --- | --- | --- |
| **Austin-Travis Lakes** |  |  |  |
|  | 1209020503 | City of Austin-Colorado River | 848.77 |
|  | 1209020504 | Onion Creek-Colorado River | 944.14 |
| **Llano** |  |  |  |
|  | 1209020402 | Big Saline Creek-Llano River | 787.56 |
|  | 1209020403 | Honey Creek-Llano River | 731.25 |
|  | 1209020404 | Little Devils River-James River | 879.60 |
|  | 1209020405 | Comanche Creek-Llano River | 958.23 |
|  | 1209020406 | Hickory Creek-Llano River | 1092.77 |
|  | 1209020407 | San Fernando Creek-Llano River | 870.56 |
|  | 1209020408 | Little Llano River-Llano River | 616.74 |
| **Lower Colorado** |  |  |  |
|  | 1209030201 | Skull Creek-Colorado River | 890.35 |
| **Lower** |  |  |  |
| **Colorado-Cummins** |  |  |  |
|  | 1209030102 | Piney Creek-Colorado River | 497.75 |
|  | 1209030104 | Alum Creek-Colorado River | 482.73 |
|  | 1209030107 | Buckners Creek-Colorado River | 1316.76 |
| **Middle Colorado** |  |  |  |
|  | 1209010606 | San Saba River-Colorado River | 758.23 |
| **North Llano** |  |  |  |
|  | 1209020202 | Middle North Llano River | 803.70 |
|  | 1209020203 | Lower North Llano River | 560.22 |
| **Pedernales** |  |  |  |
|  | 1209020601 | Headwaters Pedernales River | 1095.37 |
|  | 1209020602 | North Grape Creek-Pedernales | 1094.16 |
|  |  | River |  |
|  | 1209020603 | Pedernales River-Lake Travis | 1127.10 |
| **San Saba** |  |  |  |
|  | 1209010905 | Elm Creek-San Saba River | 534.72 |
|  | 1209010907 | Tiger Creek-San Saba River | 1043.93 |
| **South Concho** | 1209010908 | Richland Springs Creek-San Saba River | 923.85 |
|  | 1209010203 | Dove Creek | 689.75 |
| **South Llano** |  |  |  |
|  | 1209020302 | Middle South Llano River | 564.39 |
|  | 1209020304 | Lower South Llano River | 494.04 |
|  |  |  |  |

Table S3: Broad land cover classes used to reclassify historical and current land use and land cover (LULC) dataset within HUC 10 watersheds (Figure 1) within the Colorado River Basin, Texas.

Historical LULC data was re-classified from the Anderson II classification system used to classify Landsat images from the 1970’s ad 1980’s. Current LULC was reclassified from remote sensing data collected in 2011 and classified by Texas Parks and Wildlife Ecological Systems of Texas.

| **Broad Reclassed Classes** | **Historical** | **Current** |
| --- | --- | --- |
| **Agriculture** | Cropland, pasture, orchards, groves, vineyards, nurseries, ornamental horticultural, confined feeding operations, other agricultural land | Agriculture |
| **Barren** | Dry salt flats, Beaches, Sandy areas not beaches, bare exposed rock, strip mines, quarries, gravel pits, transitional areas, mixed barren land, bare ground | Barren, Cliff |
| **Deciduous** | Deciduous forest land | Deciduous Forest, Deciduous Shrubland, Floodplain CD Forest, Floodplain Deciduous Shrubland, Floodplain Live Oak Forest, Live Oak Forest, Mesquite Shrubland, Post Oak Forest, Riparian CD Forest, Riparian Deciduous Shrubland, Riparian Live Oak Forest, Sandy Oak Forest, Slope Cold Deciduous Forest, Slope Deciduous Shrubland, Slope Live Oak Forest |
| **Evergreen** | Evergreen forest land | Evergreen Shrubland, Floodplain Juniper Forest, Floodplain Juniper Shrubland, Juniper Forest, Juniper Shrubland, Pine Forest, Riparian Juniper Forest, Riparian Juniper Shrubland, Slope Evergreen Shrubland, Slope Juniper Forest |
| **Herbaceous** | Herbaceous rangeland, Shrub and Brush rangeland, Mixed rangeland, Shrub and brush tundra, Herbaceous tundra | Floodplain Herbaceous, Grassland, Marsh, Riparian Herbaceous |
| **Mixed** | Mixed forest land | Floodplain Mixed Forest, Mixed Forest, Riparian Mixed Forest, Slope Mixed Forest |
| **Water** | Streams and Canals, Lakes, Reservoirs | Open Water |
| **Urban High** | Commercial and Services, Industrial, Transportation, communication, utilities, Industrial and commercial complexes, Mixed urban or built-up land, other urban or built-up land | Urban High |
| **Urban Low** | Residential | Urban Low |
| **Wetland** | Forested wetland, Non-forested wetland | Swamp |

Table S4: Summary table of USGS gage numbers for study sites of interest. USGS gage data was accessed through USGS.

| **USGS Gage Number** | **Gage Name** |
| --- | --- |
| 08130500 | Dove Ck at Knickerbocker, TX |
| 08144500 | San Saba Rv at Menard, TX |
| 08144600 | San Saba Rv nr Brady, TX |
| 08146000 | San Saba Rv at San Saba, TX |
| 08148500 | N Llano Rv nr Junction, TX |
| 08149900 | S Llano Rv at Flat Rock Ln at Junction, TX |
| 08150000 | Llano Rv nr Junction, TX |
| 08150700 | Llano Rv nr Mason, TX |
| 08151500 | Llano Rv at Llano, TX |
| 08152900 | Pedernales Rv nr Fredericksburg, TX |
| 08153500 | Pedernales Rv nr Johnson City, TX |
| 08155240 | Barton Ck at Lost Ck Blvd nr Austin, TX |
| 08155300 | Barton Ck at Loop 360, Austin, TX |
| 08158000 | Colorado Rv at Austin, TX |
| 08158700 | Onion Ck nr Driftwood, TX |
| 08159000 | Onion Ck at US Hwy 183, Austin, TX |
| 08159200 | Colorado Rv at Bastrop, TX |
| 08159500 | Colorado Rv at Smithville, TX |
| 08160400 | Colorado Rv abv La Grange, TX |
| 08161000 | Colorado Rv at Columbus, TX |

Table S5: Catalog ID numbers for all Guadalupe Bass *Micropterus treculii* used in the morphometric analysis. These specimens were accessed at the Texas Natural History Collection in the Biodiversity Collections of the Department of Integrative Biology at The University of Texas at Austin.

*Hendrickson, Dean A. and Adam E. Cohen. 2015. Fishes of Texas Project and Online Database (version 2.0) (*[*http://fishesoftexas.org*](http://www.fishesoftexas.org/home)*). Published by the* [*Ichthyology Collection*](https://integrativebio.utexas.edu/biodiversity-collections/collections/ichthyology-fish) *of* [*The University of Texas at Austin*](http://www.utexas.edu/)*. Accessed (3 September 2015).*

| ***Catalog ID*** | ***Specimens*** | ***River*** | ***County*** | ***Location*** | ***Collecting Date*** | ***Collector*** | ***Coordinates*** |
| --- | --- | --- | --- | --- | --- | --- | --- |
| [TNHC10349](http://www.fishesoftexas.org/specimen/TNHC10349) | 6 | Llano | Llano | Llano River 1 km NE Kingsland off Highway 1431 | 18-Jul-77 | Edwards | 30.65119252, -98.48866364 |
| [TNHC10110](http://www.fishesoftexas.org/specimen/TNHC10110) | 6 | Onion Creek | Travis | Onion Creek, 12 km. SE Austin, State Hwy 183 | 19-Mar-78 | Edwards | 30.17791123, -97.68897812 |
| [TNHC10114](http://www.fishesoftexas.org/specimen/TNHC10114) | 4 | Barton Creek | Travis | Barton Creek, 21 km. SW Austin at State Hwy 71 | 9-Oct-77 | Edwards | 30.296277, -97.925624 |
| [TNHC10117](http://www.fishesoftexas.org/specimen/TNHC10117) | 34 | Onion Creek | Travis | Onion Creek, 12 km. SE Austin, State Hwy 183 | 18-Jun-77 | Edwards | 30.17791123, -97.68897812 |
| [TNHC10192](http://www.fishesoftexas.org/specimen/TNHC10192) | 42 | Llano | Llano | Llano River, 19 km. W Llano off Hwy 152 | 16-Jul-78 | Edwards | 30.71027191, -98.86003926 |
| [TNHC10194](http://www.fishesoftexas.org/specimen/TNHC10194) | 1 | Llano | Llano | Llano River, Kingsland near Kingsland Estates | 24-Apr-77 | Edwards | 30.64016489, -98.47570928 |
| [TNHC10198](http://www.fishesoftexas.org/specimen/TNHC10198) | 4 | Pedernales | Gillespie | Pedernales River, 6.4 km. S Fredericksburg, State Hwy 16 | 16-Dec-78 | Edwards | 30.20919787, -98.94879018 |
| [TNHC10200](http://www.fishesoftexas.org/specimen/TNHC10200) | 15 | Llano | Llano | Llano River, Llano near Hwy 16 | 11-Jun-78 | Edwards | 30.75243093, -98.67581467 |
| [TNHC10205](http://www.fishesoftexas.org/specimen/TNHC10205) | 2 | Llano | Llano | Llano River, Llano near Hwy 16 | 18-Nov-78 | Edwards | 30.75243093, -98.67581467 |
| [TNHC10206](http://www.fishesoftexas.org/specimen/TNHC10206) | 4 | Llano | Llano | Llano River, Llano near Hwy 17 | 18-Nov-78 | Edwards | 30.75243093, -98.67581467 |
| [TNHC10207](http://www.fishesoftexas.org/specimen/TNHC10207) | 39 | Onion Creek | Travis | Onion Creek, 12 km. SE Austin, State Hwy 183 | 4-Jul-77 | Edwards | 30.17791123, -97.68897812 |
| [TNHC10213](http://www.fishesoftexas.org/specimen/TNHC10213) | 43 | Pedernales | Travis | Pedernales River, 40 km. E Johnson City, State Hwy 962 | 21-Jun-77 | Edwards | 30.33990517, -98.13912996 |
| [TNHC10218](http://www.fishesoftexas.org/specimen/TNHC10218) | 52 | Pedernales | Gillespie | Pedernales River, 6.5 km. SE Fredericksburg, US Hwy 290 | 6-Aug-77 | Edwards | 30.22709728, -98.94879023 |
| [TNHC10225](http://www.fishesoftexas.org/specimen/TNHC10225) | 3 | Pedernales | Blanco | Pedernales River, 12 km. W Johnson City, State Hwy 1320 | 3-Dec-77 | Edwards | 30.2722066, -98.54552012 |
| [TNHC10229](http://www.fishesoftexas.org/specimen/TNHC10229) | 13 | Colorado | Colorado | Colorado River, Columbus, State Hwy 90 | 10-Jul-77 | Edwards | 29.70625864, -96.53656784 |
| [TNHC10232](http://www.fishesoftexas.org/specimen/TNHC10232) | 1 | Pedernales | Gillespie | Pedernales River, 6.5 km. SE Fredericksburg, US Hwy 290 | 5-Mar-78 | Edwards | 30.22709728, -98.94879023 |
| [TNHC10233](http://www.fishesoftexas.org/specimen/TNHC10233) | 1 | Pedernales | Gillespie | Pedernales River, State Hwy 1 Immediately Downstream from Lbj Ranch | 3-Dec-77 | Edwards | 30.2445474, -98.59765137 |
| [TNHC10239](http://www.fishesoftexas.org/specimen/TNHC10239) | 18 | Llano | Llano | Llano River, .2 km. N Castell, State Hwy 2768 | 28-Aug-76 | Edwards | 30.70380192, -98.95863175 |
| [TNHC10241](http://www.fishesoftexas.org/specimen/TNHC10241) | 13 | Llano | Llano | Llano River, Llano near Hwy 16 | 15-Oct-77 | Edwards | 30.75243093, -98.67581467 |
| [TNHC10247](http://www.fishesoftexas.org/specimen/TNHC10247) | 5 | Colorado | Colorado | Colorado River, 7.2 km. NE Altair, US Hwy 90A | 10-Jul-77 | Edwards | 29.58034302, -96.41714457 |
| [TNHC10327](http://www.fishesoftexas.org/specimen/TNHC10327) | 4 | Colorado | Travis | Colorado River, 14.5 km. E Austin, Hwy 973 | 31-Oct-76 | Edwards | 30.20818034, -97.6380769 |
| [TNHC10330](http://www.fishesoftexas.org/specimen/TNHC10330) | 2 | Llano | Kimble | Llano River, 32 km. NE Junction, State Hwy 385 | 20-Feb-77 | Edwards | 30.6587426, -99.32412093 |
| [TNHC10113](http://www.fishesoftexas.org/specimen/TNHC10113) | 46 | Llano | Mason | 14.5 km. SE Mason, State Hwy 87 | 17-Jun-77 | Edwards | 30.66117297, -99.10949548 |
| [TNHC17318](http://www.fishesoftexas.org/specimen/TNHC17318) | 1 | Dove Creek | Irion | Dove Creek at first crossing | 22-Feb-86 | Hubbs, Marsh-Matthews, and Scott | 31.15589373, -100.7527988 |
| [TNHC17355](http://www.fishesoftexas.org/specimen/TNHC17355) | 3 | Llano | Kimble | Llano River at Junction | 25-Jun-86 | Hubbs and Morales | 30.49784685, -99.75186133 |
| [TNHC17356](http://www.fishesoftexas.org/specimen/TNHC17356) | 1 | Llano | Kimble | Llano River at Junction | 25-Jun-86 | Hubbs and Morales | 30.49784685, -99.75186133 |
| [TNHC2072](http://www.fishesoftexas.org/specimen/TNHC2072) | 2 | San Saba | Menard | San Saba River, 1 mi. N Ft. McKavitt | 10-Feb-52 | Hubbs and Strawn | 30.83592528, -100.1050113 |
| [TNHC2525](http://www.fishesoftexas.org/specimen/TNHC2525) | 1 | San Saba | Menard | San Saba River, 1 mi. E Ft. McKavitt | 10-Feb-52 | Hubbs and Strawn | 30.83461535, -100.093721 |
| [TNHC2599](http://www.fishesoftexas.org/specimen/TNHC2599) | 1 | North Concho | Tom Green | N Fork Concho River, near dam, San Angelo | 9-Feb-52 | Hubbs, Strawn, Henderson, and Pyburn | 31.46714483, -100.450052 |
| [TNHC3068](http://www.fishesoftexas.org/specimen/TNHC3068) | 1 | South Llano | Kimble | S Fork Llano River, 14.5 mi. SW Junction | 27-Dec-52 | Hubbs and Strawn | 30.38543019, -99.88780445 |
| [TNHC3102](http://www.fishesoftexas.org/specimen/TNHC3102) | 3 | Dove Creek | Irion | Dove Creek, headsprings, 7 mi. SW Knickerbocker | 21-Feb-53 | Hubbs and Strawn | 31.20565229, -100.7065677 |
| [TNHC3276](http://www.fishesoftexas.org/specimen/TNHC3276) | 1 | Colorado | Bastrop | Colorado River at SH969 Southeast of Utley | 13-Mar-53 | Hubbs | 30.16746206, -97.40291082 |
| [TNHC5419](http://www.fishesoftexas.org/specimen/TNHC5419) | 1 | San Saba | Menard | San Saba River north of Fort McKavett | 16-Jul-56 | Hubbs and Strawn | 30.83592528, -100.1050113 |
| [TNHC8008](http://www.fishesoftexas.org/specimen/TNHC8008) | 1 | San Saba | Menard | San Saba River at SH 864 (first crossing north-northeast of Fort McKavett) | 16-Jul-56 | Hubbs and Strawn | 30.83461535, -100.093721 |
| [TNHC5419](http://www.fishesoftexas.org/specimen/TNHC5419) | 1 | San Saba | Menard | N Valley Prong San Saba River, 1 mi. N Ft. McKavitt | 17-Jul-56 | Hubbs and Strawn | 30.83592528, -100.1050113 |
| [TCWC235.01](http://www.fishesoftexas.org/specimen/TCWC235.01) | 3 | Colorado River | Colordao | Colorado River drainage; 6.0 mi NE Columbus on Cummings Creek | 24-Oct-59 | Jones | 29.73585769, -96.5089472 |
| [TCWC6670.03](http://www.fishesoftexas.org/specimen/TCWC6670.03) | 2 | Llano | Llano | Llano River; Llano River: 4 mi W Llano. | 30-May-86 | WFS 300 Class | 30.74395108, -98.73857625 |
| [TCWC7826.06](http://www.fishesoftexas.org/specimen/TCWC7826.06) | 5 | Dove Creek | Tom Green | Dove Creek.; Dove Creek, 0.5 mi NW Knickerbocker at FR 2335. | 21-May-91 | Brown and Smith | 31.27377036, -100.630536 |
| [TCWC8925.08](http://www.fishesoftexas.org/specimen/TCWC8925.08) | 3 | Colorado River | Concho | Colorado River; Colorado River, FM 1929 downstream from Freese Dam | 14-Oct-96 | Brown and Smith | 31.49830568, -99.66221199 |
| [TNHC10536](http://www.fishesoftexas.org/specimen/TNHC10536) | 6 | Onion Creek | Travis | State Hwy 183 crossing, 5 km. SE Austin | 6-Aug-80 | Pezold | 30.17791123, -97.68897812 |
| [TNHC11233](http://www.fishesoftexas.org/specimen/TNHC11233) | 1 | Colorado River | Travis | Colorado River near SH 71 in vicinity of Del Valle | 4-Oct-81 | Winemiller | 30.21226018, -97.64852718 |
| [TNHC21719](http://www.fishesoftexas.org/specimen/TNHC21719) | 2 | Barton Creek | Travis | Barton Creek at Lost Creek Blvd. | 18-May-93 | Warren and Freeman | 30.27412783, -97.8444023 |
| [TNHC10536](http://www.fishesoftexas.org/specimen/TNHC10536) | 6 | Onion Creek | Travis | Onion Creek, State Hwy 183 crossing, 5 km. SE Austin | 6-Aug-80 | Pezold | 30.17791123, -97.68897812 |
| [TNHC1428](http://www.fishesoftexas.org/specimen/TNHC1428) | 1 | Pedernales River | Travis | Pedernales River at Cypress Creek | 11-May-51 | Jameson and Phillips | 30.35100481, -98.13701993 |
| [TNHC21746](http://www.fishesoftexas.org/specimen/TNHC21746) | 1 | Barton Creek | Travis | Barton Creek at SH 71 | 15-Feb-93 | Warren and Wright | 30.29627697, -97.92562442 |
| [TNHC21806](http://www.fishesoftexas.org/specimen/TNHC21806) | 5 | Barton Creek | Travis | Barton Creek at Barton West subdivision off Bee Cave drive | 7-Jul-88 | Kleinsasser and Linam | 30.29889697, -97.87623317 |
| [TNHC22059](http://www.fishesoftexas.org/specimen/TNHC22059) | 1 | South Llano | Kimble | South Llano River at first FM 377 crossing SW of Junction | 21-Jun-89 | Kleinsasser and Sager | 30.36201094, -99.88930442 |
| [TNHC22323](http://www.fishesoftexas.org/specimen/TNHC22323) | 1 | Little Barton Creek | Travis | Little Barton Creek at private raod off SH 71 W of Austin near confluence with Barton Creek | 15-Mar-89 | Linam and Sauders | 30.29586698, -97.92731446 |
| [TNHC22797](http://www.fishesoftexas.org/specimen/TNHC22797) | 3 | Barton Creek | Travis | Barton Creek, 13.8 mi SW of Austin on St. Hwy. 71 | 7-Jun-93 | Warren and Freeman | 30.29627697, -97.92562442 |
| [TNHC22817](http://www.fishesoftexas.org/specimen/TNHC22817) | 79 | Barton Creek | Travis | Barton Creek, Austin, 0.8 mi E of Lost Creek Blvd. on Plumbrook Road | 11-Jul-93 | Warren and Freeman | 30.269878, -97.82928191 |
| [TNHC22819](http://www.fishesoftexas.org/specimen/TNHC22819) | 5 | Barton Creek | Travis | Barton Creek, Austin, below Barton Springs Pool at Zilker Park | 29-Jul-93 | Warren and Freeman | 30.26495827, -97.76569028 |
| [TNHC22841](http://www.fishesoftexas.org/specimen/TNHC22841) | 4 | Barton Creek | Travis | Barton Creek, Austin, 1.7 mi S of Loop 360 on Lost Creek Blvd. | 24-Aug-93 | Warren and Freeman | 30.27412783, -97.8444023 |
| [TNHC22848](http://www.fishesoftexas.org/specimen/TNHC22848) | 1 | Barton Creek | Travis | Barton Creek, Austin, below Barton Springs Pool at Zilker Park | 20-Nov-93 | Warren and Freeman | 30.26495827, -97.76569028 |
| [TNHC22866](http://www.fishesoftexas.org/specimen/TNHC22866) | 1 | Barton Creek | Travis | Barton Creek, Austin, 1.7 mi S of Loop 360 on Lost Creek Blvd. | 19-Dec-93 | Warren and Freeman | 30.27412783, -97.8444023 |
| [TNHC22915](http://www.fishesoftexas.org/specimen/TNHC22915) | 9 | Barton Creek | Travis | Barton Creek. 1.4 mi S of Bee Cave Road on Crystal Creek Drive | 17-Jun-94 | Warren and Freeman | 30.30145691, -97.86433287 |
| [TNHC22928](http://www.fishesoftexas.org/specimen/TNHC22928) | 30 | Barton Creek | Travis | Barton Creek, 1.4 mi SW of Loop 360 on Plumbrook road below Lost Creek subdivision | 28-Aug-93 | Warren, Freeman, and Hiers | 30.269878, -97.82928191 |
| [TNHC22973](http://www.fishesoftexas.org/specimen/TNHC22973) | 1 | Little Barton Creek | Travis | Little Barton Creek at private road off SH 71 W of Austin at Fandango Way | 7-Jul-88 | Kleinsasser and Linam | 30.29586698, -97.92731446 |
| [TNHC22989](http://www.fishesoftexas.org/specimen/TNHC22989) | 1 | Barton Creek | Travis | Barton Creek, 1.4 mi SW of Loop 360 on Plumbrook road below Lost Creek subdivision | 25-Aug-93 | Warren and Freeman | 30.269878, -97.82928191 |
| [TNHC230](http://www.fishesoftexas.org/specimen/TNHC230) | 5 | Onion Creek | Travis | 10 mi. SE on Onion Creek, near del Valle | 17-Sep-47 | Blair | 30.18925098, -97.6201864 |
| [TNHC23004](http://www.fishesoftexas.org/specimen/TNHC23004) | 7 | Barton Creek | Travis | Barton Creek, 13.8 mi SW of Austin on St. Hwy. 71 | 23-Aug-93 | Warren and Freeman | 30.29627697, -97.92562442 |
| [TNHC23015](http://www.fishesoftexas.org/specimen/TNHC23015) | 2 | Barton Creek | Travis | Barton Creek, 7.0 mi NE of Dripping Springs on Co. Rd. 185 (or Trautwein Road) | 24-Aug-93 | Warren and Freeman | 30.23644873, -98.0248168 |
| [TNHC23116](http://www.fishesoftexas.org/specimen/TNHC23116) | 7 | Colorado River | Bastrop | Colorado River in town of Bastrop at loop 150 river crossing | Jul-87 | Morales | 30.10989408, -97.32286866 |
| [TNHC23120](http://www.fishesoftexas.org/specimen/TNHC23120) | 1 | Colorado River | Bastrop | Colorado River in town of Bastrop at loop 150 river crossing | Oct-86 | Morales | 30.10989408, -97.32286866 |
| [TNHC23122](http://www.fishesoftexas.org/specimen/TNHC23122) | 8 | Colorado River | Bastrop | Colorado River in town of Bastrop at loop 150 river crossing | Jun-87 | Morales | 30.10989408, -97.32286866 |
| [TNHC23146](http://www.fishesoftexas.org/specimen/TNHC23146) | 1 | Colorado River | Bastrop | Colorado River in town of Bastrop at loop 150 river crossing | Nov-86 | Morales | 30.10989408, -97.32286866 |
| [TNHC23155](http://www.fishesoftexas.org/specimen/TNHC23155) | 7 | Colorado River | Bastrop | Colorado River at Pope Bend off of FM 969 approx 7 mi E of its jct with SH71 | May-86 | Morales | 30.18786136, -97.42345139 |
| [TNHC23163](http://www.fishesoftexas.org/specimen/TNHC23163) | 3 | Colorado River | Bastrop | Colorado River at Pope Bend off of FM 969 approx 7 mi E of its jct with SH71 | Jul-87 | Morales | 30.18786136, -97.42345139 |
| [TNHC23173](http://www.fishesoftexas.org/specimen/TNHC23173) | 3 | Colorado River | Bastrop | Colorado River at Pope Bend off of FM 969 approx 7 mi E of its jct with SH71 | Sep-86 | Morales | 30.18786136, -97.42345139 |
| [TNHC23181](http://www.fishesoftexas.org/specimen/TNHC23181) | 1 | Colorado River | Bastrop | Colorado River at Pope Bend off of FM 969 approx 7 mi E of its jct with SH71 | August -September 1986 | Morales | 30.18786136, -97.42345139 |
| [TNHC23196](http://www.fishesoftexas.org/specimen/TNHC23196) | 15 | Colorado River | Bastrop | Colorado River at Pope Bend off of FM 969 approx 7 mi E of its jct with SH71 | Jun-87 | Morales | 30.18786136, -97.42345139 |
| [TNHC23203](http://www.fishesoftexas.org/specimen/TNHC23203) | 1 | Colorado River | Travis | Colorado River at FM 973 highway crossing | Sep-86 | Morales | 30.20816034, -97.6379969 |
| [TNHC23233](http://www.fishesoftexas.org/specimen/TNHC23233) | 2 | Colorado River | Travis | Colorado River at Longhorn Dam | May-86 | Morales | 30.25036883, -97.71344891 |
| [TNHC23244](http://www.fishesoftexas.org/specimen/TNHC23244) | 3 | Colorado River | Travis | Colorado River at Longhorn Dam | Jun-87 | Morales | 30.25036883, -97.71344891 |
| [TNHC23605](http://www.fishesoftexas.org/specimen/TNHC23605) | 5 | Colorado River | Colorado | Colorado River at Smithville from about 50m below St. Hwy. 95 to 400m below St. Hwy. 71 | 29-Jun-96 | Hendrickson, Mosier, and Southwest Texas State University Class | 30.02294702, -97.26388697 |
| [TNHC23645](http://www.fishesoftexas.org/specimen/TNHC23645) | 4 | Colorado River | Travis | Colorado River at FM 973 highway crossing | Jul-87 | Morales | 30.20816034, -97.6379969 |
| [TNHC23648](http://www.fishesoftexas.org/specimen/TNHC23648) | 2 | Colorado River | Travis | Colorado River at FM 973 highway crossing | Jul-87 | Morales | 30.20816034, -97.6379969 |
| [TNHC23651](http://www.fishesoftexas.org/specimen/TNHC23651) | 2 | Colorado River | Travis | Colorado River at Longhorn Dam | Jun-87 | Morales | 30.25036883, -97.71344891 |
| [TNHC23658](http://www.fishesoftexas.org/specimen/TNHC23658) | 3 | Colorado River | Bastrop | Colorado River at Pope Bend off of FM 969 approx 7 mi E of its jct with SH71 | August -September 1986 | Morales | 30.18786136, -97.42345139 |
| [TNHC2525](http://www.fishesoftexas.org/specimen/TNHC2525) | 1 | San Saba | Menard | San Saba River at SH 864 (first crossing north-northeast of Fort McKavett) | 10-Feb-52 | Hubbs and Strawn | 30.83461535, -100.093721 |
| [TNHC2599](http://www.fishesoftexas.org/specimen/TNHC2599) | 1 | N Fork Concho River | Tom Green | North Concho River downstream of O. C. Fisher Dam | 9-Feb-52 | Hubbs, Strawn, Henderson, and Pyburn | 31.46714483, -100.450052 |
| [TNHC29916](http://www.fishesoftexas.org/specimen/TNHC29916) | 3 | Walnut Creek | Travis | Walnut Creek, reach from Springdale Road upstream to Sprinkle Cutoff Road | 20-Mar-03 | Hendrickson, Hendrickson, and Hicks | 30.35145566, -97.65306761 |
| [TNHC29926](http://www.fishesoftexas.org/specimen/TNHC29926) | 3 | Walnut Creek | Travis | Walnut Creek, reach from Springdale Road upstream to Sprinkle Cutoff Road | 30-Mar-03 | Hendrickson, Hendrickson, and Hicks | 30.35145566, -97.65306761 |
| [TNHC29947](http://www.fishesoftexas.org/specimen/TNHC29947) | 3 | Walnut Creek | Travis | Walnut Creek, reach from Springdale Road upstream to Sprinkle Cutoff Road | 30-Mar-03 | Hendrickson, Hendrickson, and Hicks | 30.35145566, -97.65306761 |
| [TNHC5076](http://www.fishesoftexas.org/specimen/TNHC5076) | 1 | Pedernales River | Travis | Pedernales River at confluence of Cypress Creek and Hamilton Creek | 6-May-55 | McCoy | 30.34975485, -98.13725993 |
| [TNHC538](http://www.fishesoftexas.org/specimen/TNHC538) | 2 | Colorado River | Travis | Colorado River at Waller Street | 3-Oct-47 | Blair and Class | 30.25068878, -97.73535947 |
| [TNHC7313](http://www.fishesoftexas.org/specimen/TNHC7313) | 1 | Llano | Llano | Llano River, near Submerged bridge Past Kingsland to The Left of Fr 1431 | 8-Mar-68 | Rogers and Leach | 30.68206354, -98.4841796 |
| [TNHC8200](http://www.fishesoftexas.org/specimen/TNHC8200) | 1 | Llano | Kimble | Llano River at Junction | 28-Apr-68 | Eddleman | 30.49784685, -99.75186133 |

Table S6: Canonical structure coefficients for the first three canonical variates for relative warp scores used in the canonical correlation analysis of Guadalupe Bass *Micropterus treculii* morphology related to altered flow and land cover throughout the Colorado River basin, Texas. Historical individuals were collected by Edwards (1980) and stored at the Texas Natural Historical Museum collection where morphological photos were taken for analysis (Table S4). Present-day individuals were collected between March 2014 and September 2016. Location of landmarks comprising relative warps are illustrated in Figure 3. Bolded values indicate the most correlated variables for the particular morphological variable.

| **Variable** | **morphology1** | **morphology2** |
| --- | --- | --- |
| **RW1** | **-0.9358** | -0.0647 |
| **RW2** | **0.3221** | 0.0253 |
| **RW3** | 0.0682 | **-0.9797** |
| **RW4** | 0.0686 | **0.2135** |

Table S7: Canonical structure coefficients for the first three canonical variates for environmental variables (land use and landcover (LULC) changes and indicators of hydrologic alteration (IHA) used in the canonical correlation analysis of Guadalupe Bass *Micropterus treculii* morphology related to altered flow and LULC throughout the Colorado River basin, Texas. Historical individuals were collected by Edwards (1980) and stored at the Texas Natural Historical Museum collection where morphological photos were taken for analysis (Table S4) Present-day individuals were collected between March 2014 and September 2016. Location of landmarks comprising relative warps are illustrated in Figure 3. Bolded values indicate the most correlated variables for the particular morphological variable.

| Variable | **Environment 1** | **Environment 2** |
| --- | --- | --- |
| Herbaceous | **0.6782** | -0.0738 |
| Barren | 0.2873 | 0.335 |
| Reversals | -0.0237 | **0.4426** |
| 0 Flow Days | **0.3997** | **0.4912** |
| 7-Day Max | **0.5264** | 0.2509 |
| 30-Day Max | **0.4017** | **0.3919** |
| Baseflow | -0.2404 | **-0.2579** |
| 3-Day Max | **0.3725** | 0.1466 |
| Forested | **-0.5197** | 0.0854 |
| 90-Day Max | **0.445** | **0.3848** |
| Fall Rate | 0.2696 | -0.0207 |
| 7-Day Min | -0.3672 | 0.082 |
| Low Pulse Count | **-0.4104** | **0.4601** |
| November | 0.0172 | -0.0114 |
| Agriculture | -0.0028 | 0.0427 |
| Date of Minimum | 0.2782 | 0.2662 |
| December | -0.1988 | 0.0404 |
| 30-Day Min | 0.0773 | 0.1674 |
| High Pulse Count | **0.3944** | 0.039 |

**
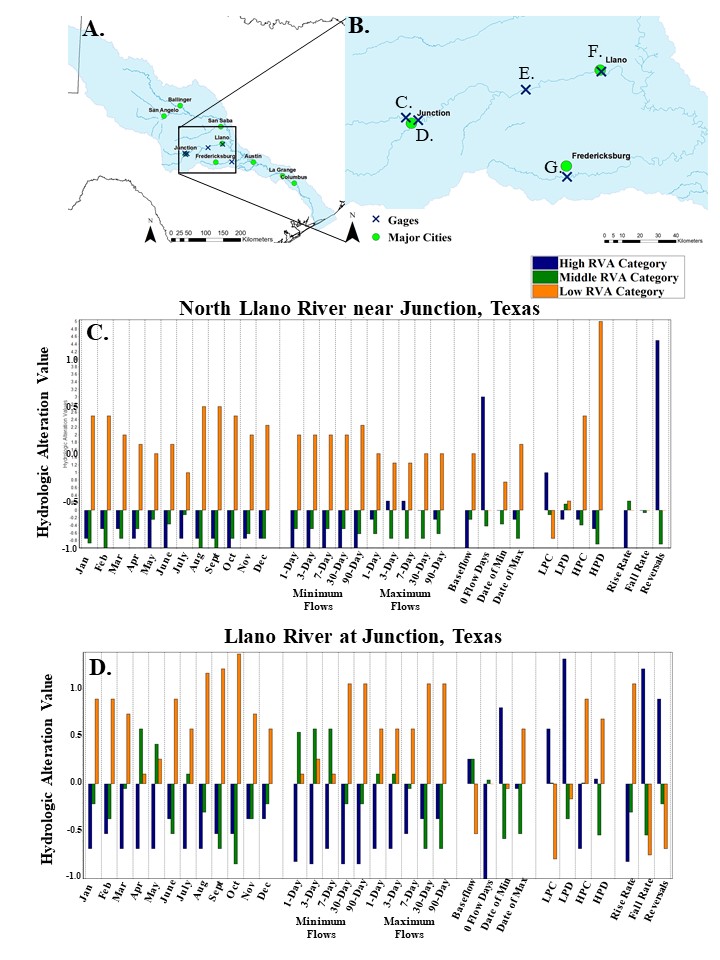

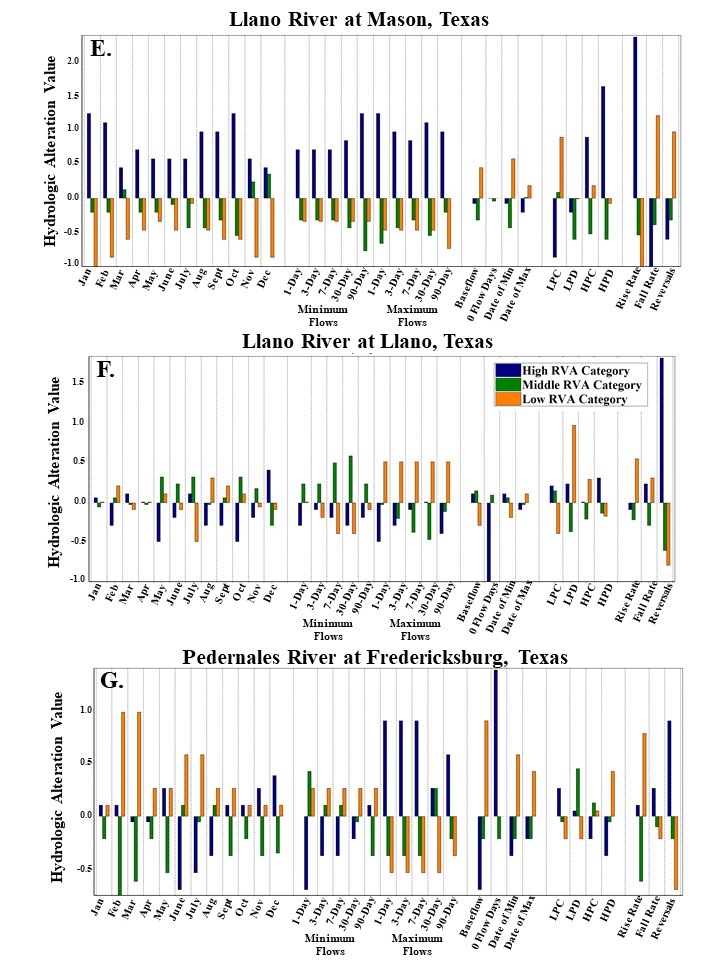
**

Figure S1: Map and bar graphs representing hydrologic alteration between pre-1980 and post-1995 for the U.S. Geological Survey (USGS) gaging stations closest to the sampling locations on the Llano River and Pedernales River in Texas with historic and current discharge records.

Hydrologic alteration was assessed using the range of variability approach (RVA) as described by Richter et al. (1997). RVA scores have a maximum value of infinity and a minimum value of -1. The 33^rd^ and 67^th^ percentiles are used to determine the three distinct categories (High, Medium, Low). Positive values indicate an increase in the frequency; negative values indicate a decrease in the frequency of the parameter values between the two-time periods within a given category. Map A and B represent the location of the stream gages (Gauges:08148500 North Llano River near Junction, Texas (C),08150000 Llano River Near Junction, Texas (D), 08150700 Llano River near Mason, Texas (E), 08151500 Llano River at Llano, Texas (F), and 08152900 Pedernales River near Fredericksburg, Texas (G)) used to determine the flow conditions an individual Guadalupe Bass *Micropterus treculii*experienced.


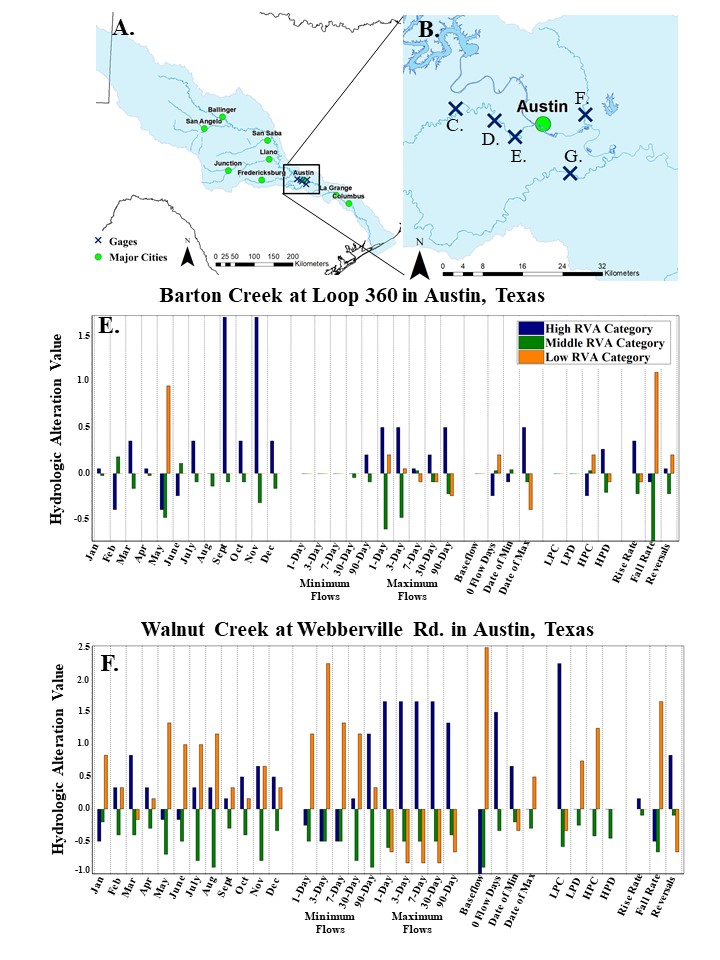


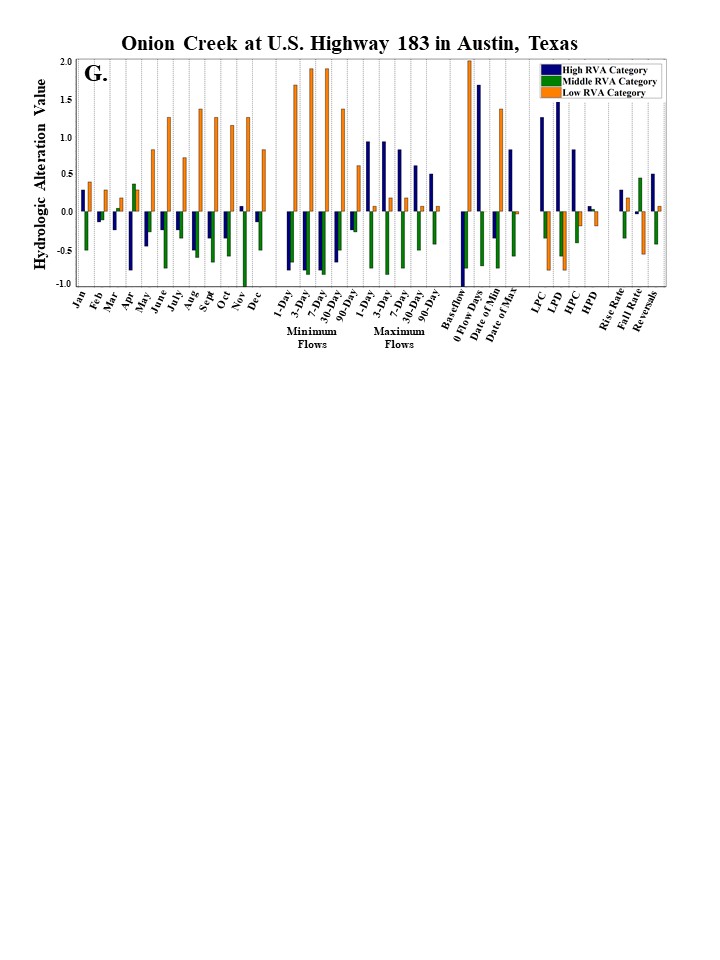


Figure S2: Map and bar graphs representing hydrologic alteration between pre-1980 and post-1995 for the U.S. Geological Survey (USGS) gaging stations closest to the sampling locations on the Llano River and Pedernales River in Texas with historic and current discharge records. Hydrologic alteration was assessed using the range of variability approach (RVA) as described by Richter et al. (1997). RVA scores have a maximum value of infinity and a minimum value of -1. The 33^rd^ and 67^th^ percentiles are used to determine the three distinct categories (High, Medium, Low). Positive values indicate an increase in the frequency; negative values indicate a decrease in the frequency of the parameter values between the two-time periods within a given category. Map A and B represent the location of the stream gages (Gauges: 08155200 Barton Creek at State Highway 71 near Oak Hill, Texas (C), 08155240 Barton Creek at Lost Creek Boulevard near Austin, Texas (D), 08155300 Barton Creek at Loop 360, Austin, Texas (E), 08159000 Onion Creek at U.S. Highway 183, Austin, Texas (F), 08158600 Walnut Creek at Webberville Road, Austin, Texas (G)) used to determine the flow conditions an individual Guadalupe Bass *Micropterus treculii* experienced. Historical discharge records were not sufficient enough to conduct RVA analysis for Barton Creek at Lost Creek or Barton Creek at State Highway 71, therefore RVA analysis is not shown for these gages.

**
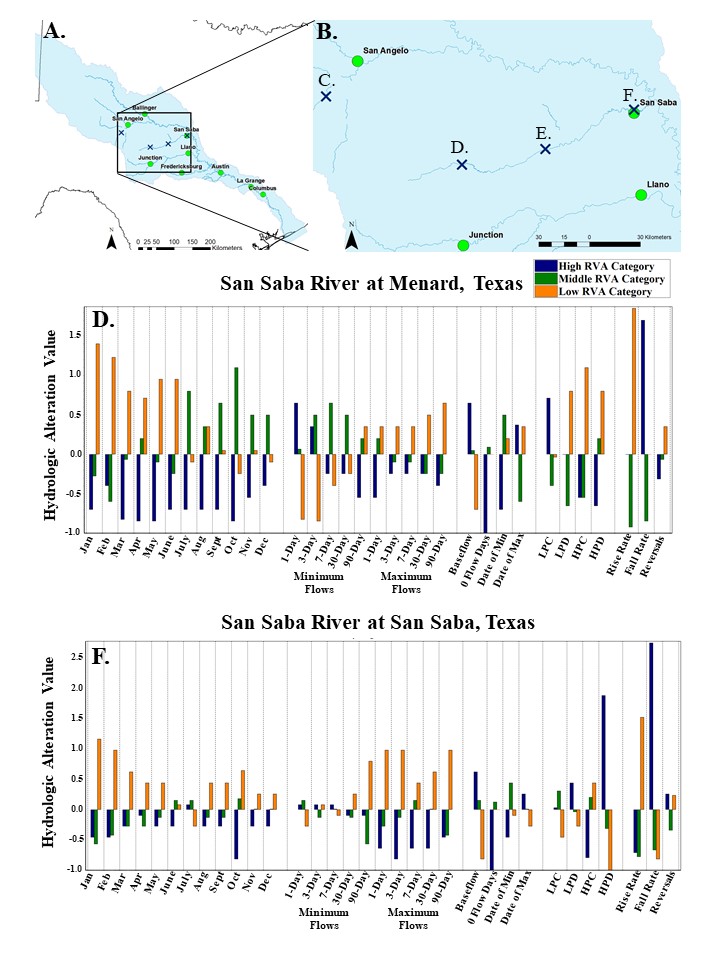
**

Figure S3: Map and bar graphs representing hydrologic alteration between pre-1980 and post-1995 for the U.S. Geological Survey (USGS) gaging stations closest to the sampling locations on the San Saba River in Texas with historic and current discharge records.

Hydrologic alteration was assessed using the range of variability approach (RVA) as described by Richter et al. (1997). RVA scores have a maximum value of infinity and a minimum value of -1. The 33^rd^ and 67^th^ percentiles are used to determine the three distinct categories (High, Medium, Low). Positive values indicate an increase in the frequency; negative values indicate a decrease in the frequency of the parameter values between the two-time periods within a given category. Map A and B represent the location of the stream gages (Gauges: 08144500 San Saba River at Menard, Texas (C), 08144600 San Saba River near Brady (D), Texas, 08146000 San Saba at San Saba, Texas (E)) used to determine the flow conditions an individual Guadalupe Bass *Micropterus treculii* experienced. Historical discharge records were not sufficient enough to conduct RVA analysis for the San Saba River at Brady, Texas, therefore RVA analysis is only shown for the San Saba River at Menard, Texas (C) and for the San Saba River at San Saba, Texas (E).

**
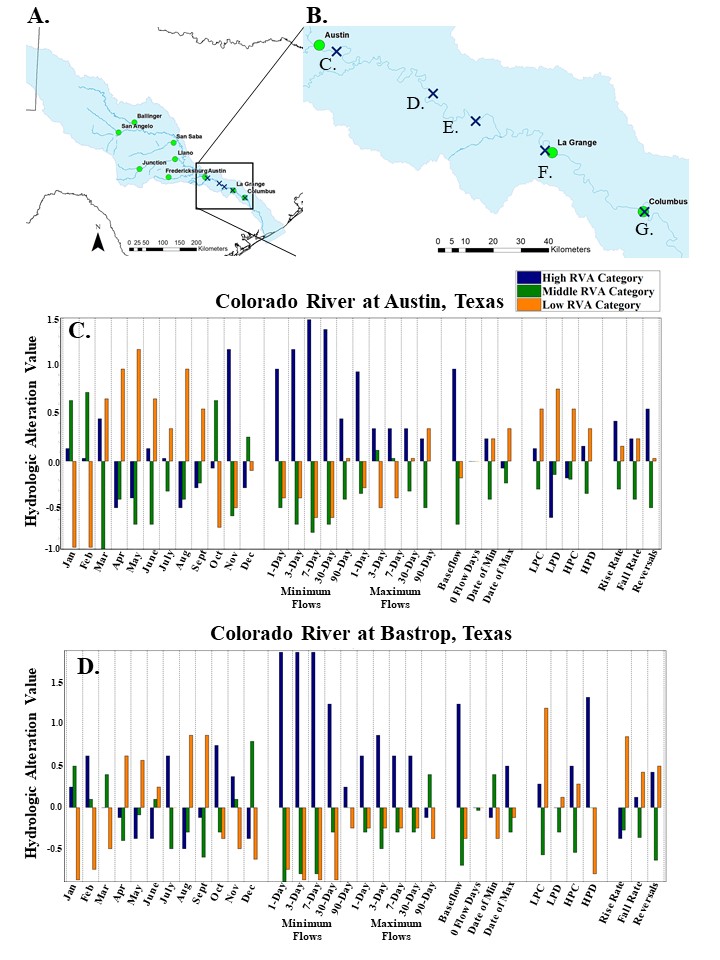

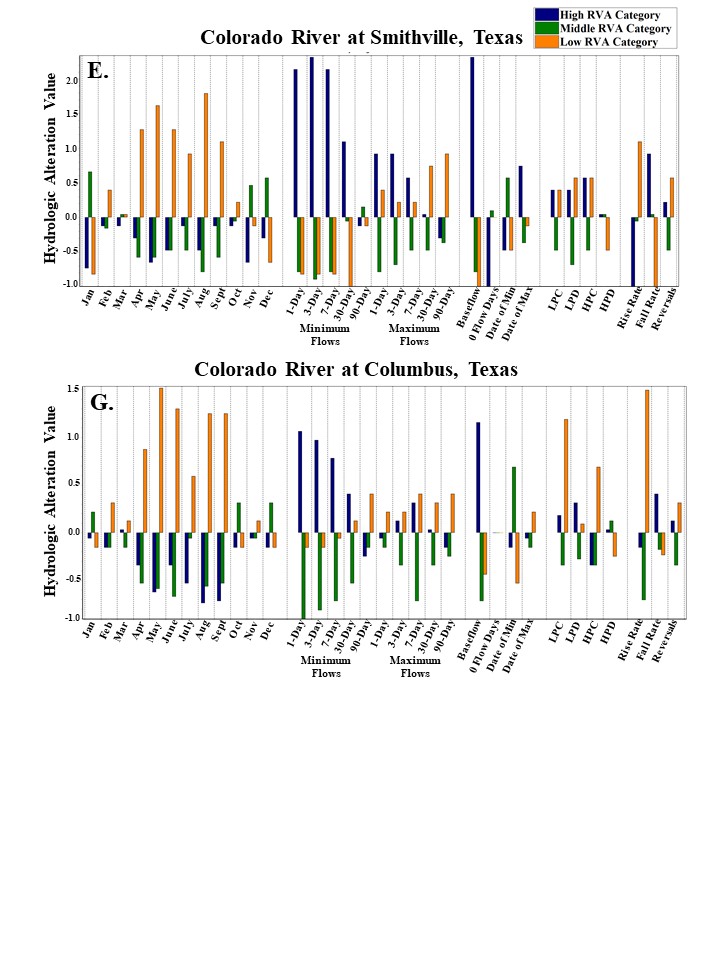
**

Figure S4: Map and bar graphs representing hydrologic alteration between pre-1980 and post-1995 for the U.S. Geological Survey (USGS) gaging stations closest to the sampling locations on the lower Colorado River in Texas with historic and current discharge records. Hydrologic alteration was assessed using the range of variability approach (RVA) as described by Richter et al. (1997). RVA scores have a maximum value of infinity and a minimum value of -1. The 33^rd^ and 67^th^ percentiles are used to determine the three distinct categories (High, Medium, Low). Positive values indicate an increase in the frequency; negative values indicate a decrease in the frequency of the parameter values between the two-time periods within a given category. Map A and B represent the location of the stream gages (Gauges:08158000 Colorado River at Austin, Texas (C), 08159200 Colorado River at Bastrop, Texas (D), 8159500 Colorado River at Smithville, Texas (E), 8160400 Colorado River at LaGrange, Texas (F), 8161000 Colorado River at Columbus, Texas (G)) used to determine the flow conditions an individual Guadalupe Bass *Micropterus treculii* experienced. Historical discharge records were not sufficient enough to conduct RVA analysis for the Colorado River at La Grange, Texas, therefore RVA analysis is not shown for this gage.


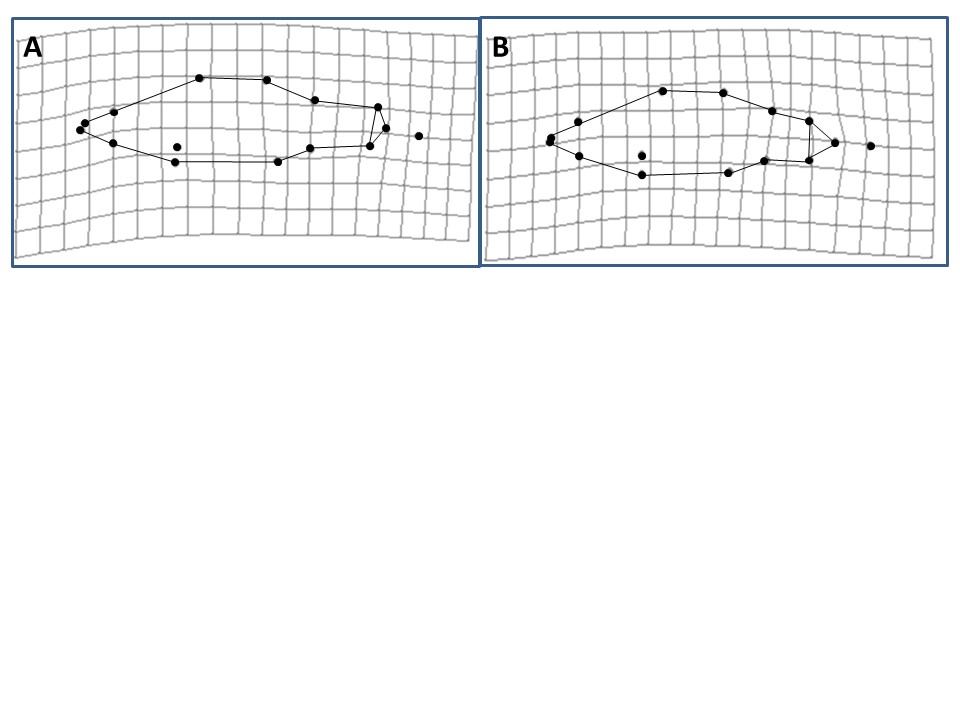


Figure S5: Thin-plate splines representing morphological variation between the average or consensus shape of Guadalupe Bass *Micropterus treculii* initial capture morphology (A) and following 18-month period of preservation in formalin for 23 individual Guadalupe Bass.

1. *Current address: U.S. Geological Survey, Hawaii Cooperative Fishery Research Unit, University of Hawaii at Hilo, Hilo, Hawaii 96720; e-mail: tgrabowski@usgs.gov [↑](#footnote-ref-1)
